# Genome-wide identification of cannabinoid biosynthesis genes in non-drug type *Cannabis* (*Cannabis sativa* L.) cultivar

**DOI:** 10.1186/s42238-024-00246-8

**Published:** 2024-09-07

**Authors:** Benny Jian Rong Sng, Yu Jeong Jeong, Sing Hui Leong, Jae Cheol Jeong, Jiyoung Lee, Sarojam Rajani, Cha Young Kim, In-Cheol Jang

**Affiliations:** 1 Temasek Life Sciences Laboratory, 1 Research Link, National University of Singapore, Singapore, 117604 Singapore; 2 Department of Biological Sciences, National University of Singapore, Singapore, 117543 Singapore; 3 Biological Resource Center, Korea Research Institute of Bioscience and Biotechnology, Jeongeup, 56212 Korea

**Keywords:** *Cannabis sativa*, Hemp, RNA-seq

## Abstract

**Background:**

*Cannabis sativa* cultivars can be classified as marijuana or hemp, depending on its amount of the psychoactive cannabinoid Δ^9^‐tetrahydrocannabinolic acid (THCA). Hemp Cheungsam is a non-drug type *Cannabis sativa* that is characterized by low THCA content. However, the transcripts and expression profile of cannabinoid biosynthesis pathway genes of hemp Cheungsam have not been investigated.

**Methods:**

RNA-sequencing (RNA-seq) was performed on three different tissue types (flower, leaf, and stem) of hemp Cheungsam to understand their transcriptomes. The expression of cannabinoid biosynthesis pathway genes was further analyzed in each tissue type. Multiple sequence alignment and conserved domain analyses were used to investigate the homologs of cannbinoid biosynthesis genes.

**Results:**

We found that the cannabinoid biosynthesis pathway was mainly expressed in the flowers of hemp Cheungsam, similar to other *Cannabis* cultivars. However, expression of cannabidiolic acid (CBDA) synthase was much higher than THCA synthase and cannabichromenic acid (CBCA) synthase, suggesting that the transcription profile favors CBDA biosynthesis. Sequence analysis of cannabinoid biosynthesis pathway genes suggested the identity of orthologs in hemp Cheungsam.

**Conclusions:**

Cannabinoid biosynthesis in hemp Cheungsam mostly occurs in the flowers, compared to other plant organs. While CBDA synthase expression is high, THCA and CBCA synthase expression is considerably low, indicating lesser THCA biosynthesis and thus low THCA content. Sequence analysis of key genes (CBDA, THCA, and CBCA synthases) of the cannabinoid biosynthetic pathway indicates that orthologs are present in hemp Cheungsam.

**Supplementary Information:**

The online version contains supplementary material available at 10.1186/s42238-024-00246-8.

## Introduction

Cannabis is a widely cultivated plant with a long history dating back more than 6000 years (Atakan 2012; Tahir et al. 2021). The *Cannabis* genus is composed of three species (*Cannabis sativa*, *Cannabis indica*, and *Cannabis ruderalis*), with varying levels of specific cannabinoids [Cannabidiolic acid (CBDA), Δ^9^‐tetrahydrocannabinolic acid (THCA), and cannabichromenic acid (CBCA)] depending on the species and variety (Atakan 2012; Tahir et al. 2021). Furthermore, with extensive interbreeding between species, the chemical composition of a Cannabis plant cannot be easily determined based on its morphology alone (Tahir et al. 2021). *Cannabis* can be further classified into marijuana or hemp, depending on the amount of psychoactive cannabinoid THCA (Hilderbrand 2018; Hussain et al*.*, 2021). As marijuana has a higher THCA content, it has been cultivated for use as a recreational and medicinal drug (Hussain et al., 2021). Hemp, which has lower THCA content, was cultivated for food and industrial purposes, including the production of hemp seeds and hemp oil, textiles, and even biodegradable plastics (Cerino et al. 2021; Hussain et al., 2021).

While *C. sativa* can produce more than 180 different cannabinoid compounds, the three most abundant cannabinoids, THCA, CBDA, and CBCA have been well documented (Tahir et al. 2021). The cannabinoid biosynthetic pathway stems from hexanoic acid, which is produced from the oxidative cleavage of other fatty acids (Gülck and Møller 2020). Hexanoic acid then undergoes a multistep conversion to olivetolic acid (OLA), which is one of two main substrates for cannabinoid biosynthesis (Gülck and Møller 2020; Tahir et al. 2021). The other substrate is geranyl pyrophosphate (GPP), a methylerythritol 4-phosphate (MEP) pathway intermediate that is formed by GPP synthase (GPS) catalyzing the condensation of dimethylallyl pyrophosphate and isopentenyl pyrophosphate (Gülck and Møller 2020; Tahir et al. 2021). Aromatic prenyltransferases (PT) catalyze the conversion of OLA and GPP to cannabigerolic acid (CBGA), which is further modified to other cannabinolic acids (CBDA, THCA, CBCA) by specific synthases (Gülck and Møller 2020; Tahir et al. 2021). CBDA, THCA, and CBCA can then undergo non-enzymatic decarboxylation to form cannabidiol, Δ^9^‐tetrahydrocannabinol, and cannabichromene (CBD, THC, and CBC), respectively (Tahir et al. 2021).

As the differentiation between marijuana and hemp is based on THCA content, a previous study has investigated THCA synthase polymorphisms (and thus the presence of active THCA synthase) as a main factor for identifying marijuana and hemp plants (Roman et al. 2022). However, this was not a completely accurate method for predicting THCA content in each tested cultivar (Roman et al. 2022). This can be possibly explained by a complex ancestry of interbreeding and introgression between Cannabis cultivars. Furthermore, gene duplication and deletion during the breeding process may have affected THCA production. In another study, the CBDRx cultivar was shown to be from a primary marijuana lineage but had a CBDA synthase (CBDAS) gene from hemp and no THCA synthase (THCAS) gene (Grassa et al. 2021).

Here, we investigated the transcriptome of non-drug type hemp “Cheungsam”, which is a hybrid between the local variety of Korean hemp and the IH3 hemp cultivar from the Netherlands (Moon et al. 2002). Hemp Cheungsam is a predominant hemp variety in Korea, as its lower THCA content makes it a preferrable *C. sativa* variety for Korean traditional medicine (Moon et al. 2002; Doh et al*.*, 2019). Hemp Cheungsam samples were dissected into three different tissue types (flower, leaf, and stem) to better understand the transcriptome and cannabinoid biosynthetic pathway in various parts of the plant. We showed that similar to other *Cannabis* cultivars, cannabinoid biosynthesis genes in hemp Cheungsam were mostly expressed in the flowers. Multiple sequence alignment and conserved domain analyses also verified that the identified transcripts were mostly full-length homologs of the cannabinoid biosynthesis pathway genes.

## Materials and methods

### Plant growth conditions

*Cannabis sativa* L. seeds (Cheungsam) were soaked in 1% hydrogen peroxide solutions as liquid germination media. After one day, a fresh 1% H_2_O_2_ solution was added after the removal of the old solution. Seeds were soaked for three more days at room temperature again in the dark. Germinated seedlings were transplanted from the H_2_O_2_ solutions to soil and transferred to a growth chamber (26 ± 1 ℃, 16 h light:8 h dark cycle, 51% humidity, and light intensity of 258 µmol·m^−2^·sec^−1^). Hemp Cheungsam plants were grown in long day (LD) condition (16 h light:8 h dark) during the early stages of vegetative growth for up to 3 weeks. Subsequently, in the later vegetative growth stage, the light period was increased to 18 h light:6 h dark for another 8 to 10 weeks. To induce a transition to the reproductive stage, the photoperiod was reduced to 12 h light:12 h dark for approximately 5 weeks.

### Extraction of RNA and RNA-sequencing (RNA-seq)

Branches of hemp Cheungsam with fully developed female flowers were harvested. All branches were immediately dissected after harvesting to obtain the leaf, stem, and flower samples. The developed cola with female flowers were collected as the flower sample. Palmate leaves from each branch were consolidated as the leaf sample. The stem sample comprised of the dissected branch without any leaf or flower tissue. All samples were flash frozen in liquid nitrogen, then ground into a fine powder for RNA extraction.

Total RNA was extracted from all samples and purified using RNeasy Plant Mini Kit (Qiagen, Germany) according to the manufacturer’s instructions, including the optional on-column DNase digestion step (Qiagen, Germany). The purity and concentration of total RNA were determined using a Nanodrop spectrophotometer (DS-11 spectrophotometer, DeNovix, USA). Only RNA samples with A260/280 ratios between 1.8 and 2.2, and A260/230 ratios higher than 2.0 were kept for RNA-seq. RNA-seq was performed by Macrogen (Korea) using the manufacturer's reagents and protocol.

The RNA-seq was performed with paired-end sequencing with 101 base pair (bp) read length. RNA library was constructed using TruSeq Stranded Total RNA Library Prep Plant Kit (Illumina, USA). The samples were sequenced using NovaSeq6000 system with flow cell type S4 (Illumina, USA) at Macrogen (Korea).

The raw sequence data of the RNA-seq was firstly subjected to a quality check using FastQC (version 0.11.7, Andrews 2010). After which, the adaptor sequences were trimmed via the Trimmomatic (version 0.38, Bolger et al. 2014). Bases at the rear ends with base quality < 3 were trimmed. In addition, sliding window trimming with window size = 4 was used to remove bases with mean quality < 15. Subsequently, the trimmed sequences that were < 36 bp were also removed from further analysis.

Trimmed reads were mapped to the *Cannabis sativa* reference genome *cs10* (https://www.ncbi.nlm.nih.gov/datasets/genome/GCF_900626175.2/), using HISAT2 (version 2.1.0, Kim et al. 2019). Spliced read mapping was performed through Bowtie2 aligner (version 2.3.4.1, Langmead and Salzberg 2012). Transcript assembly onto the *cs10* reference genome was done using StringTie (version 2.1.3b, Kovaka et al. 2019), to obtain the expression profile per sample.

### Identification of differentially expressed genes (DEGs) and hierarchical clustering

Firstly, the transcriptome data was filtered to remove genes with FPKM < 1 for all samples. To calculate fold-change, 0.001 was added to all FPKM values. The average FPKM per tissue type was calculated and used to calculate gene expression fold-change between different plant tissues. DEGs were identified by fold-change > 2 or < 0.5 and Student’s *t*-test *P*-value < 0.05. Volcano plot for each pairwise comparison were generated using MATLAB version R2020a (The MathWorks Inc., USA).

DEGs identified from each pairwise comparison were plotted in a Venn diagram using the Interactivenn webtool (Heberle et al. 2015; http://www.interactivenn.net/). DEGs from the intersection of each Venn diagram were compiled and their respective FPKM values were plotted into a hierarchical clustering heatmap using MATLAB version R2020a (The MathWorks Inc., USA). Gene clusters identified from hierarchical clustering were used for further bioinformatics analysis.

### Bioinformatics analysis for gene ontology (GO) terms and Kyoto Encyclopedia of Genes and Genomes (KEGG) pathways

Protein sequences associated with each DEG were used as the query for protein Basic Local Alignment Search Tool (BLASTP) searches, which were carried out locally using BLAST + (Camacho et al. 2009). BLASTP searches were against Arabidopsis protein sequences from the TAIR10 database (Lamesch et al*.*, 2012). The output with the lowest e-value was chosen as the Arabidopsis best-fit ortholog of the hemp Cheungsam gene. Ortholog genes were filtered for e-value < 0.05 to remove results of low confidence.

The respective Arabidopsis ortholog genes were then used to identify GO terms and enriched KEGG pathways using the Database for Annotation, Visualization, and Integrated Discovery (DAVID) analysis (Huang et al. 2009; Sherman et al. 2022). GO term and KEGG pathway information were plotted in Excel.

### Multiple sequence alignment and sequence analysis

Multiple sequence alignments and generation of phylogenetic trees were performed on MegAlign 15 (DNASTAR, Inc., USA). Alignment was carried out using the Clustal W tool on MegAlign 15. Protein domains were identified using Batch CD-Search (Marchler-Bauer and Bryant, 2004; Marchler-Bauer et al., 2011; https://www.ncbi.nlm.nih.gov/Structure/bwrpsb/bwrpsb.cgi) and MOTIF search tool (GenomeNet, https://www.genome.jp/tools/motif/). Prediction of PTS1 sequence in protein sequences was performed using the PTS1 predictor (Neuberger et al. 2003; https://mendel.imp.ac.at/pts1/). The C-terminal amino acid sequence (last 12 residues) of each protein sequence was used as the input for PTS1 prediction. Prediction of protein subcellular localization was carried out using WoLF PSORT (Horton et al., 2007; https://wolfpsort.hgc.jp/).

## Results and discussion

### Pedigree, significance, and morphology of hemp ‘Cheungsam’

Hemp Cheungsam is a variety of hemp (*C. sativa*) that originated from Korea (Moon et al. 2002). The variety was developed as a hybrid between the Korean local variety hemp and IH3 hemp from the Netherlands (Moon et al. 2002). As a predominant hemp variety in Korea, hemp Cheungsam has relatively low THCA is to CBDA content, resulting it to be a classified as a non-drug type *Cannabis* (Moon et al. 2002).

As the cultivation and consumption of *Cannabis* plants are highly regulated (Ransing et al. 2022), the development of *Cannabis* varieties is likely to be geographically restricted. Thus, in Korea, hemp Cheungsam is preferred over other local *C. sativa* varieties for its use in Korean traditional herbal medicine, due to its lower THCA content (Doh et al., 2019). The seeds and sprouts of hemp Cheungsam were also reported to contain compounds that are beneficial for human health such as Quercetin and Rutin, which have antioxidant and anti-inflammatory properties (Aloo et al., 2023a; Aloo et al., 2023b). Furthermore, CBDA from hemp Cheungsam has been used to enhance the anti-cancer activity of cabozantinib against hepatocellular carcinoma (Jeon et al. 2023).

**Fig. 1:**
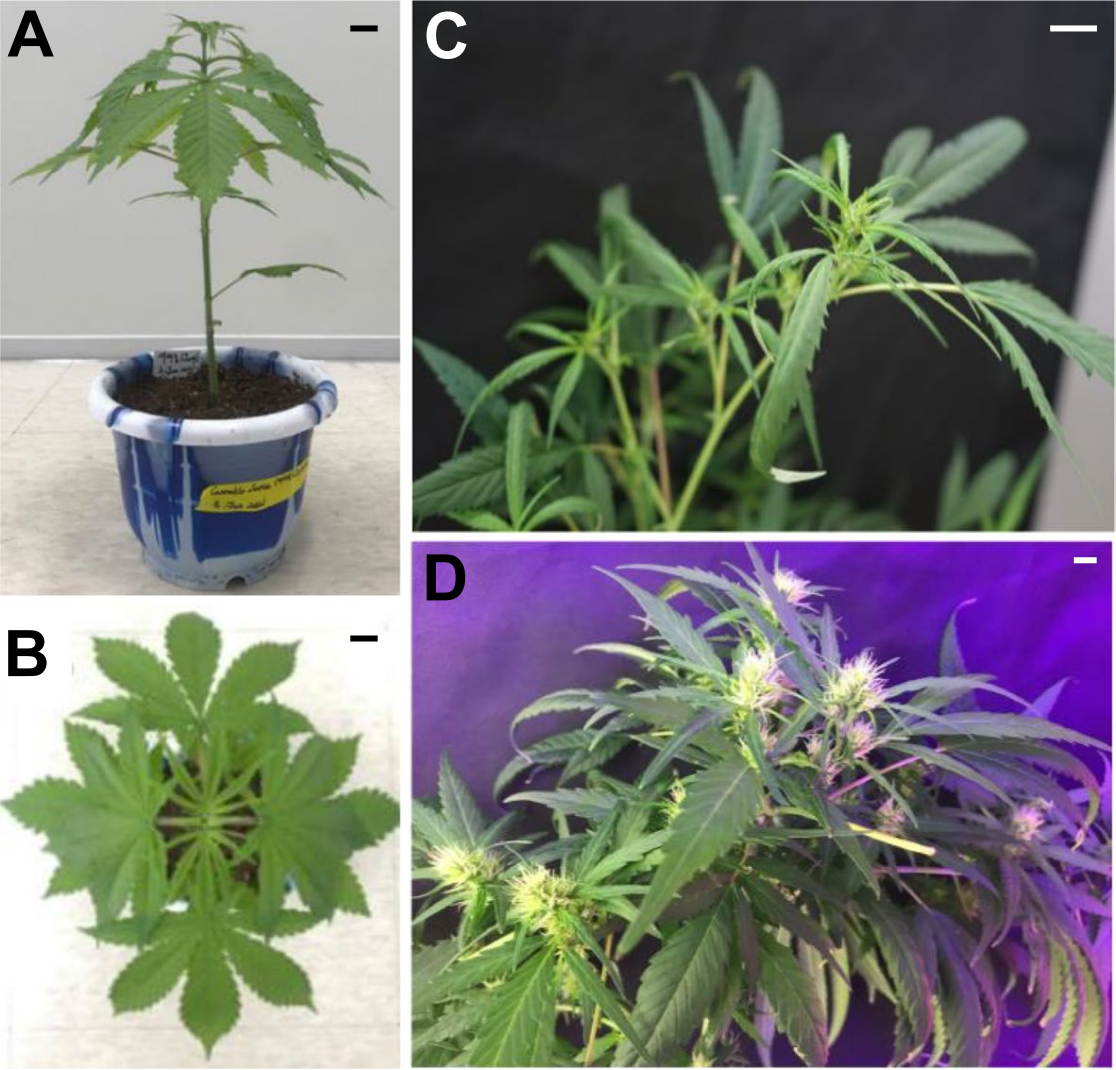
Phenotype of hemp Cheungsam. **A** Hemp Cheungsam during the vegetative growth stage. **B** Top-down view of hemp Cheungsam, showing palmate leaves. **C** Female flowers developing at the cola at 2 w after flower induction. **D** Developed female flowers at the cola at 5 w after flower induction. Black (**A**, **B**) and white (**C**, **D**) scale bars, 1 cm

Hemp Cheungsam typically undergoes 10 to 13 weeks of vegetative growth. After which, the plant transitions to the flowering induction stage of 3 to 4 weeks. Hemp Cheungsam developed palmately compound leaves along its stem (Fig. 1A), which is similar to other varieties of *C. sativa* (Anderson and de la Paz 2021). Each compound leaf is made of green pinnate leaflets with serrated leaf margin (Fig. 1B). At the reproductive stage, hemp Cheungsam developed cola with female flowers, which started developing approximately 2 weeks after flower induction (Fig. 1C) and were fully developed at 5 weeks after flower induction (Fig. 1D).

### RNA-seq analysis of flower, leaf, and stem tissues of hemp Cheungsam

To understand the effects of gene transcription on its physiology and production of cannabinoids in different tissues of hemp Cheungsam, mature hemp plants with female flowers were dissected into flower, leaf, and stem tissues for RNA-seq analysis (Fig. 1). After removing adapter sequences and trimming low quality reads, all RNA-seq samples had more than 97% clean reads (Table 1). In addition, high Phred quality scores indicated high sequencing quality, as Q20 scores were above 97.8% and Q30 scores were above 93.3% in all samples (Table 1).

**Table 1:** Summary of RNA-seq reads

Sample		Total reads	Clean reads	GC (%)	Q20 (%)	Q30 (%)
Flower						
Replicate 1		76,227,318	74,237,116	44.36	98.08	93.86
Replicate 2		71,180,562	69,652,226	44.68	98.82	95.82
Replicate 3		65,076,956	63,640,172	44.61	98.78	95.71
Leaf						
Replicate 1		71,336,918	69,508,966	41.13	97.88	93.38
Replicate 2		65,078,200	63,559,016	41.90	98.79	95.78
Replicate 3		63,037,978	61,667,536	41.91	98.72	95.53
Stem						
Replicate 1		88,895,932	86,601,516	42.49	98.09	93.86
Replicate 2		69,761,540	68,156,156	42.38	98.69	95.44
Replicate 3		76,021,312	74,357,434	42.76	98.85	95.87

**Fig. 2:**
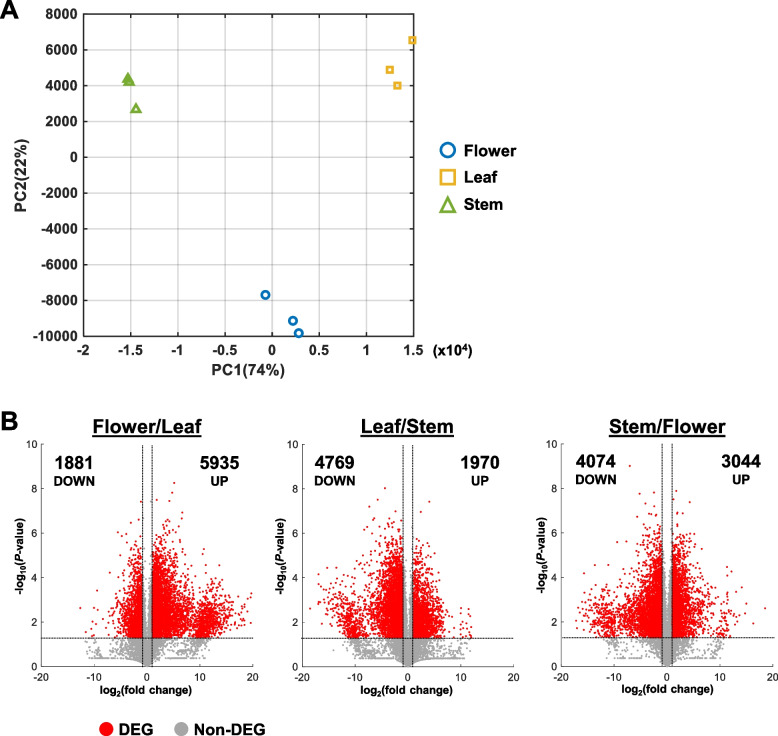
Comparison of transcriptomes between different tissue types. **A** Principal component analysis (PCA) plot of sample triplicates. PC1, principal component 1. PC2, Principal component 2. **B** Volcano plots for all sample comparisons. FPKM values were compared between two samples. Each volcano plot shows the distribution of fold change and Student’s t-test for all transcripts. Differentially expressed genes (DEGs) were identified with fold change > 2 or fold change < 0.5 and *P*-value < 0.05. Number of up- and down-regulated DEGs are indicated in each plot

Reproducibility between replicates was verified by the Pearson correlation coefficient between samples (Fig. S1). Replicates of each tissue type showed a high correlation coefficient between replicates and a lower correlation between tissue types (Fig. S1). Similarly, samples from each tissue type formed distinct clusters in the PCA plot, suggesting that the transcriptomes of flower, leaf, and stem tissues differ greatly from each other (Fig. 2A). Furthermore, while all three tissue types varied in PC1, flower samples had distinct PC2 values from leaf and stem samples (Fig. 2A).

### Identification of differentially expressed genes (DEGs)

Genes with FPKM < 1 in all samples were excluded from further analysis due to their extremely low expression. To identify DEGs, pairwise comparison of FPKM values were carried out between the various plant tissues. DEGs were identified by filtering for genes with fold change > 2 or < 0.5 and *P*-value < 0.05 (Table S1). In addition, the fold change and *P*-value of all genes were visualized as volcano plots to better identify the transcriptomic differences between samples (Fig. 2B).

Interestingly, the most up-regulated DEGs were identified in the Flower/Leaf comparison with 5935 genes (Fig. 2B; Table 2). In comparison, the least up-regulated DEGs belonged to the Leaf/Stem comparison with 1970 genes (Fig. 2B; Table 2). In contrast, the most down-regulated DEGs were identified in the Leaf/Stem comparison with 4769 genes while the least down-regulated DEGs were from the Flower/Leaf comparison with 1881 genes (Fig. 2B; Table 2).

**Table 2:** Number of up- and down-regulated DEGs in comparisons between different tissue types

Number of DEGs	Flower/Leaf comparison	Leaf/Stem comparison	Stem/Flower comparison
Up-regulated	5935	1970	3044
Down-regulated	1881	4769	4074

### Identification of DEGs with specifically high- or low-expression in each plant tissue type

**Fig. 3:**
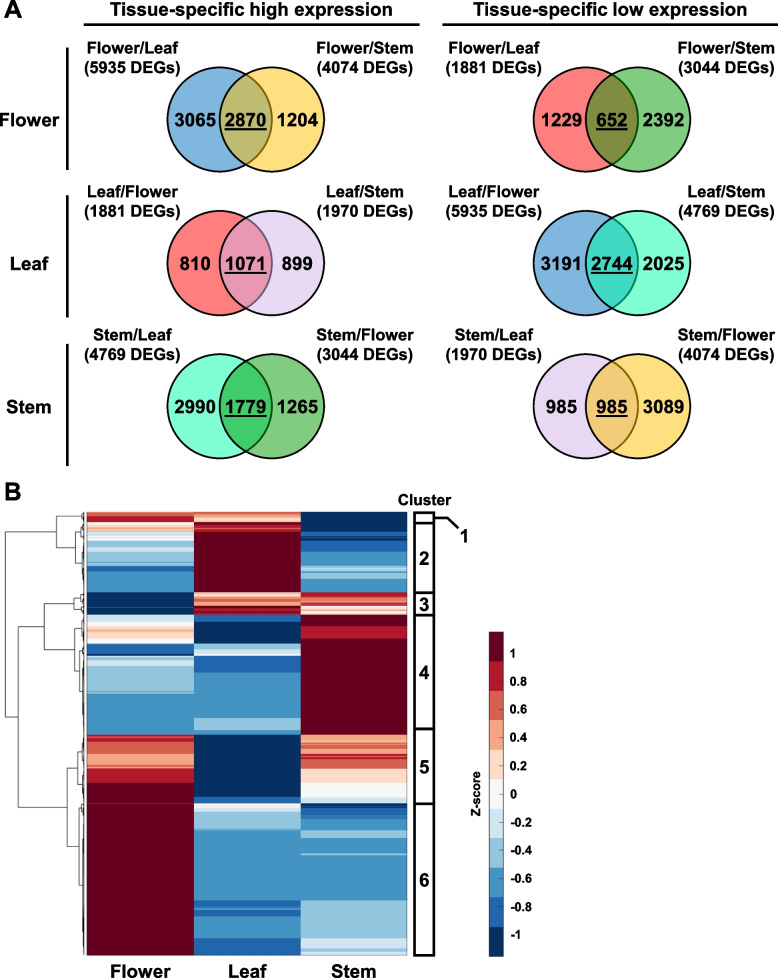
Identification of tissue-specific genes in hemp Cheungsam flower, leaf, and stem. **A** Venn diagrams show overlap of DEGs identified from all comparisons. DEGs shared between two comparisons are underlined. **B** Hierarchical clustering heatmap of shared DEGs, showing expression pattern of tissue-specific genes. Red color represents high expression while blue color represents low expression

As there were three different hemp tissues in this analysis, pairwise comparisons would be unable to directly identify genes that are specifically induced or repressed in one specific tissue type. To address this limitation, the DEGs identified from each pairwise comparison were plotted into Venn diagrams. The shared DEGs in each Venn diagram represent genes that are specifically induced or repressed in each tissue type, as compared to the other plant tissues (Fig. 3A, Table S2). From this analysis, we found that flowers had the most DEGs with tissue-specific high expression and the least DEGs with tissue-specific low expression (Fig. 3A). On the other hand, leaf samples had the least DEGs with high expression and most DEGs with low expression (Fig. 3A). This corroborates with a previous study, which also showed that female *Cannabis* flowers have more up-regulated DEGs than other plant organs (Braich et al. 2019).

The expression pattern of these tissue-specific DEGs was visualized in a hierarchical clustering heat map, which revealed six gene clusters (Fig. 3B, Table S3). Gene cluster 1 was enriched in the flower and leaf (Fig. 3B). Cluster 2 was specifically induced in leaf samples, while cluster 3 was up-regulated in both leaf and stem samples (Fig. 3B). Cluster 4 was enriched in only the stem, but cluster 5 was enriched in both flower and stem (Fig. 3B). Cluster 6 which is the largest gene cluster was highly expressed in only the flower samples (Fig. 3B). The highly expressed genes in flower samples may be attributed to the abundance of trichomes on female flowers, as a previous study showed overlapping expression profiles between female flowers and trichomes (Braich et al. 2019).

### Tissue-specific enrichment of GO terms and KEGG pathways

Although our RNA-seq is aligned to the *Cannabis sativa* reference genome *cs10* and other Cannabis reference genomes are available (van Bakel et al. 2011; Cai et al. 2021), GO term analysis of Cannabis genes is not readily available. As such, we performed a BLASTP search of each DEG’s protein sequence against the Arabidopsis TAIR10 database to identify close orthologs of each hemp Cheungsam gene. E-value < 0.05 was used to ensure high homology in identifying the Arabidopsis ortholog (Table S4). Identification of Arabidopsis orthologs was successful for most DEGs, as more than 85% of DEGs per gene cluster were mapped to an Arabidopsis ortholog (Table 3, Table S4). Furthermore, gene cluster 6 had the largest number of DEGs with no Arabidopsis orthologs (No Hit, Table 3), indicating that flower samples may express more genes that are unique to *Cannabis*.

**Table 3:** Number of orthologs identified per gene cluster

Number of DEGs	Cluster 1	Cluster 2	Cluster 3	Cluster 4	Cluster 5	Cluster 6
TAIR10 ortholog	187 (93.5%)	1219 (92.9%)	365 (85.1%)	2043 (90.6%)	1208 (93.7%)	2674 (93.3%)
No hit	0	13	8	34	15	44
Pseudogene	8	33	14	64	47	86
lncRNA	3	30	29	93	19	51
snoRNA	1	8	13	15	0	5
snRNA	1	1	0	2	0	4
tRNA	0	9	0	4	0	2
Total DEGs	200	1312	429	2255	1289	2866

**Fig. 4:**
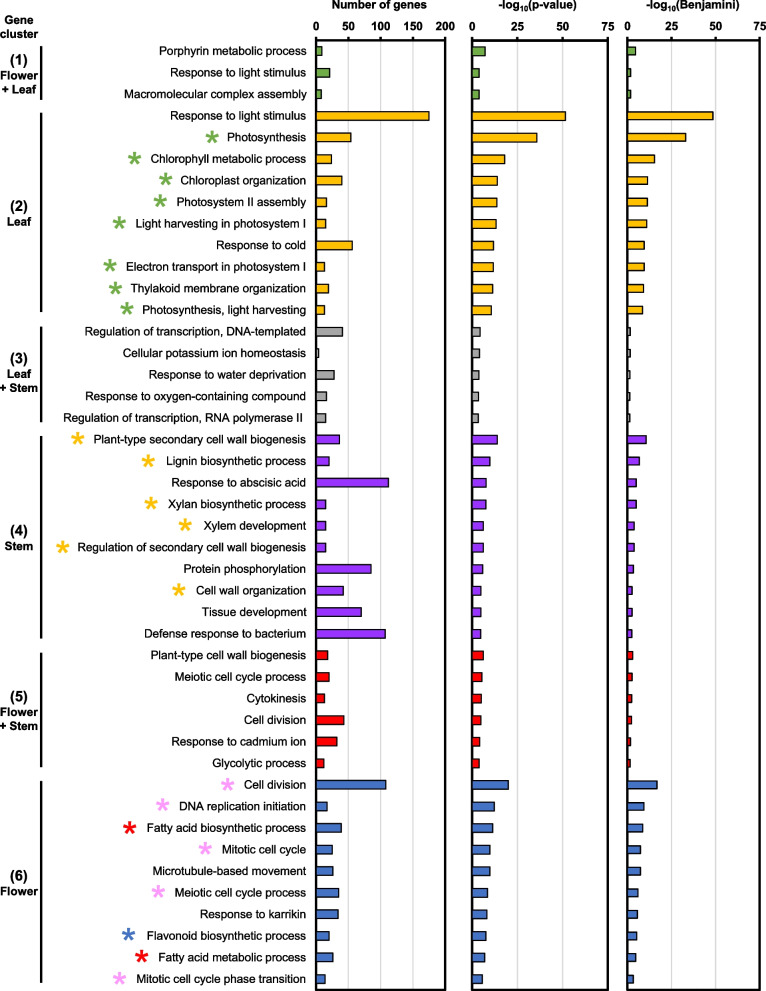
Enriched biological process (BP) GO terms in various plant tissue types. GO terms were selected to have Benjamini–Hochberg adjusted *P*-value (Benjamini) < 0.05. Top 10 GO terms are shown. Gene clusters correspond to Fig. 3B

The Arabidopsis orthologs are subsequently used to identify biological process (BP) GO terms that are specifically induced in each gene cluster (Table S5). Gene cluster 2 consists of DEGs that were highly expressed in leaf samples, which corresponded with GO terms related to photosynthesis and chloroplasts, such as “photosynthesis” and “chlorophyll metabolic process” (green stars, Fig. 4). In contrast, gene cluster 4 comprises of DEGs with high expression in stem samples and were enriched in GO terms related to plant cell wall and vasculature development, including “plant-type secondary cell wall biogenesis” and “xylem development” (yellow stars, Fig. 4). Importantly, gene cluster 6 contains DEGs that were enriched in flowers and were related to GO terms involved in fatty acid biosynthesis and metabolism (red stars, Fig. 4). GO terms related to cell division were also identified in cluster 6, such as “meiotic cell cycle process” and “mitotic cell cycle” (pink stars, Fig. 4). Interestingly, the “flavonoid biosynthetic process” GO term was also found to be specific to flower samples (blue star, Fig. 4). While GO terms were also identified in gene clusters 1, 3, and 5, which were expressed in multiple tissue types, their enrichment was less significant (Fig. 4).

Furthermore, the Arabidopsis orthologs were used to identify KEGG pathways from each gene cluster (Table S6). Leaf-specific DEGs in gene cluster 2 were associated with KEGG pathways related to photosynthesis like “photosynthesis – antenna proteins” and “carbon fixation in photosynthetic organisms” (green stars, Fig. S2). Interestingly, stem-specific DEGs in cluster 4 were related to “phenylpropanoid biosynthesis” and “stilbenoid, diarylheptanoid, and gingerol biosynthesis” (yellow stars, Fig. S2). DEGs in gene cluster 5, which were up-regulated in the flower and stem, were involved in amino acid metabolism such as “biosynthesis of amino acids” and “alanine, aspartate, and glutamate metabolism” (light blue stars, Fig. S2). Moreover, KEGG pathways related to “carbon metabolism” and “glycolysis/gluconeogenesis” were also identified in cluster 5 (pink stars, Fig. S2). Lastly, KEGG pathways that are specific to flower samples in gene cluster 6 were related to “fatty acid biosynthesis/metabolism” (red stars) as well as “flavonoid biosynthesis” (blue star, Fig. S2). Conversely, gene clusters 1 (up-regulated in flower and leaf) and 3 (up-regulated in leaf and stem) did not correlate to any biologically meaningful KEGG pathway (Fig. S2).

### Analysis of cannabinoid biosynthetic pathway in each tissue type of hemp Cheungsam

While both GO term and KEGG pathway analyses identified fatty acid biosynthesis and metabolism to be specifically enriched in flowers of hemp Cheungsam, the analyses did not identify cannabinoid biosynthesis. This can be explained by the lack of Arabidopsis orthologs for cannabinoid biosynthetic pathway genes and the relative exclusivity of cannabinoid biosynthesis to a small number of organisms. While cannabinoid biosynthesis was initially thought to be specific to *C. sativa*, it is now known that other plants, such as *Rhododendron dauricum* and *Radula marginata*, also have cannabinoid biosynthetic pathway genes (Gülck and Møller 2020).

**Fig. 5:**
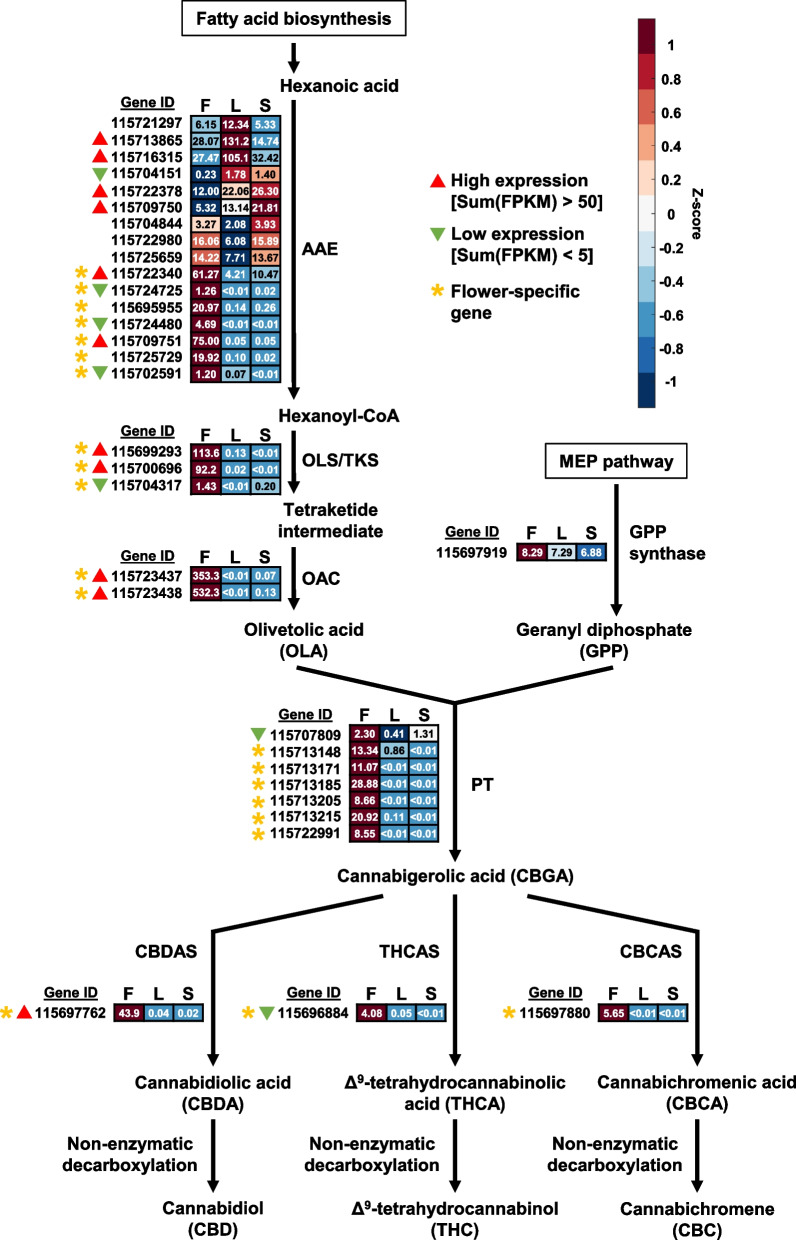
Expression of full-length cannabinoid biosynthetic pathway genes in various hemp Cheungsam plant tissues. Expression levels are represented by both Z-score and FPKM value. Red color indicates high Z-score while blue color indicates low Z-score. Each row represents a gene homolog. The number in each box represents FPKM value. Sum(FPKM) refers to total FPKM from flower, leaf, and stem samples. Flower-specific gene is defined as expression in flowers samples being more than five-fold than leaf or stem samples. AAE, ACYL-ACTIVATING ENZYME. OLS/TKS, OLIVETOL SYNTHASE/TETRAKETIDE SYNTHASE. OAC, OLIVETOLIC ACID CYCLASE. PT, AROMATIC PRENYLTRANSFERASE. CBDAS, CBDA synthase. THCAS, THCA synthase. CBCAS, CBCA synthase. F, flower. L, leaf. S, stem

In hemp Cheungsam, many of the cannabinoid biosynthetic pathway genes were specifically expressed in the flowers (Fig. 5, Table S7). These include orthologs of genes encoding acyl-activating enzyme (AAE), olivetol synthase (OLS)/tetraketide synthase (TKS), olivetolic acid cyclase (OAC), and aromatic prenyltransferase (PT), which are responsible for generating the precursors for cannabinoid production (Fig. 5). Interestingly, 7 out of the 16 *AAE* orthologs in hemp Cheungsam were specifically expressed in the flowers as compared to other tissues (Fig. 5), indicating that these *AAEs* are crucial for cannabinoid biosynthesis. In contrast, the *C. sativa* marijuana cultivar Purple Kush displayed similar expression levels of *AAE1* and *AAE3* in stems, flowers, and other plant tissues (van Bakel et al. 2011). This suggests that gene expression patterns in hemp Cheungsam may differ from other varieties of *C. sativa*. The expression of all downstream genes *OLS/TKS*, *OAC*, *PT*, and *CBDA/THCA/CBCA synthases* (*CBDAS*, *THCAS*, and *CBCAS*) were highly specific to the flowers, indicating that cannabinoid biosynthesis occurs mostly in the flowers of hemp Cheungsam (Fig. 5). This is consistent with the transcriptome of *C. sativa* Purple Kush (van Bakel et al. 2011) and in agreement with the knowledge that cannabinoid biosynthesis predominantly occurs in the glandular trichomes of female flowers of *Cannabis* (Zager et al. 2019).

While most cannabinoid biosynthetic pathway genes are specific to flowers, we noted that the overall expression levels may vary between orthologs. For example, *OLS/TKS* orthologs *115699293* and *115700696* have high FPKM values, while unigene *115704317* has low expression even in the flowers (Fig. 5). This suggests that unigenes *115699293* and *115700696* are likely to be the main *OLS/TKS* orthologs in hemp Cheungsam. By comparing the total FPKM value across all tissue types (flower, leaf, and stem), orthologs with high and low expression levels were identified (Fig. 5). Orthologs with high expression were found for *AAE*, *OLS/TKS*, and *OAC*, while *GPS* showed moderate expression (Fig. 5). In contrast, all 7 *PT* showed either moderate or low expression (Fig. 5). Downstream of CBGA, only the *CBDAS* ortholog showed high expression (Fig. 5). In contrast, the *THCAS/CBCAS* orthologs displayed moderate or low expression (Fig. 5). Taken together, this suggests that cannabinoid biosynthesis in hemp Cheungsam favors CBDA production over THCA and CBCA, which is consistent with a previous report showing high CBDA and low THCA content (Moon et al. 2002).

### Analysis of cannabinoid biosynthetic pathway genes

#### Acyl-activating enzyme (AAE)

**Fig. 6:**
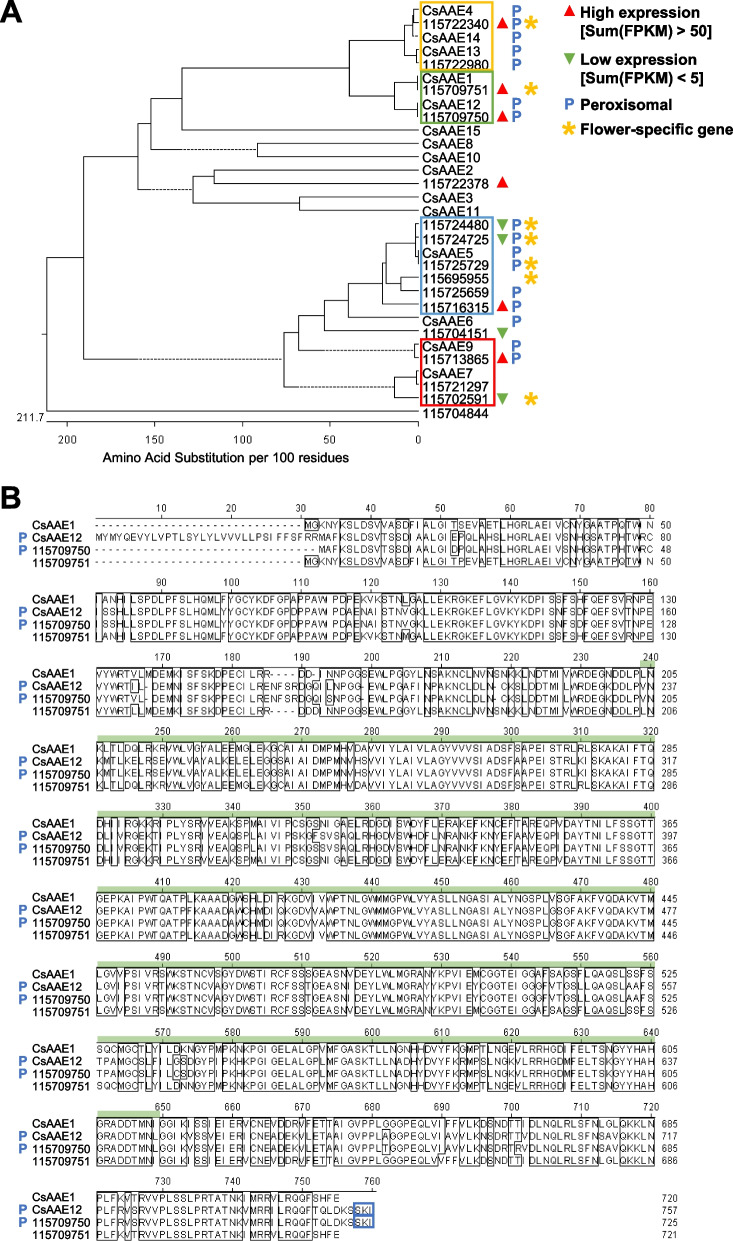
AAE orthologs in hemp Cheungsam. **A** Phylogenetic tree of hemp Cheungsam and GenBank database AAE protein sequences. Solid lines indicate actual phylogenetic distance. Dotted lines are used to align all terminals and do not represent phylogenetic distance. Colored boxes indicate four main groups of AAE identified in the phylogenetic tree. Sum(FPKM) refers to total FPKM from flower, leaf, and stem samples. **B** Multiple protein sequence alignment of CsAAE1 and CsAAE12 with hemp Cheungsam orthologs. Green bar, AMP-binding domain. Blue boxes highlight C-terminus peroxisome targeting signal type 1 (PTS1; Reumann 2004). Blue P indicates predicted peroxisomal AAE

The synthesis of cannabinoids begins with the conversion of hexanoic acid to short-chain fatty acyl-coenzyme A (CoA) precursor hexanoyl-CoA by AAE (Stout et al. 2012). Hemp Cheungsam has 16 *AAE* gene orthologs, which showed high protein homology to known *AAE* genes from the GenBank database (Fig. 6A). Only unigene *115704844* was phylogenetically distant from the other *AAE* genes (Fig. 6A). *CsAAE1* was previously suggested to be a hexanoyl-CoA synthase involved in the cannabinoid biosynthetic pathway, based on its high expression in glandular trichomes, hexanoyl-CoA synthase activity, and subcellular localization to the cytoplasm (Stout et al. 2012). As the subsequent step (polyketide biosynthesis) in the cannabinoid biosynthetic pathway occurs in the cytoplasm, AAEs that localize to the peroxisome are implied to be involved in peroxisomal β-oxidation (Shockey et al., 2003; De Azevedo Souza et al., 2008; Stout et al. 2012).

As hemp Cheungsam unigene *115709751* showed high protein sequence similarity to *CsAAE1*, we performed multiple sequence alignment to compare their protein sequences with the nearest homologs *CsAAE12* and unigene *115709750* (green box, Fig. 6A). Multiple sequence alignment revealed that *CsAAE1*, *CsAAE12*, *115709750*, and *115709751* shared high homology in the AMP-binding domain (Fig. 6B), which is required to activate the carboxylic acid substrate (e.g. hexanoate) to form adenylate as an acyl-AMP intermediate (Shockey and Browse 2011). In addition to their high similarity, both *CsAAE12* and *115709750* have the previously reported C-terminus peroxisome targeting signal type 1 (PTS1) sequence (blue boxes, Fig. 6B; Reumann 2004; Stout et al. 2012), suggesting that they localize to the peroxisome. In contrast, *115709751* does not and likely localizes to the cytoplasm, similar to CsAAE1 (Stout et al. 2012). The presence of PTS1 was further confirmed using the PTS1 predictor (Neuberger et al. 2003), while the subcellular localization was further verified using WoLF PSORT prediction (Horton et al., 2007).

We further analyzed the multiple sequence alignments amongst each group of *AAE* homologs (yellow, blue, red boxes, Fig. 6A). Multiple sequence alignments showed that the other *AAE* groups contain the AMP-binding domain and AMP-binding C-terminal domain (Fig. S3, S4, S5). The C-terminus PTS1 sequence and peroxisomal localization was also identified in *CsAAE4*, *CsAAE13*, *CsAAE14*, *115722340*, and *115722980* (Fig. S3), which corroborates with a previous study showing *CsAAE13* and *CsAAE14* having the PTS1 sequence (Stout et al. 2012). As for homologs of *CsAAE5*, all homologs except *115695955* were predicted to contain the PTS1 sequence and localize to the peroxisome (Fig. S4). Lastly, the comparison between *CsAAE7*, *CsAAE9*, *115721297*, *115702591*, and *115713865* revealed that both *CsAAE9* and *115713865* contain the PTS1 sequence for the peroxisome localization (Fig. S5). As for other *AAE* homologs, only *CsAAE6* was predicted to have both PTS1 and peroxisomal localization (Fig. 6A). Taken together, the results imply that *115709751* is the major *AAE* ortholog in hemp Cheungsam, with high expression that is specific to the flowers and no predicted peroxisomal localization (Fig. 6A).

#### Olivetol synthase (OLS)/tetraketide synthase (TKS)

Hexanoyl-CoA undergoes sequential condensation with three malonyl-CoA, which is catalyzed by OLS/TKS to form a linear tetraketide intermediate followed by further conversion to OLA or olivetol depending on the presence or absence of OAC, respectively (Kearsey et al. 2020).

**Fig. 7:**
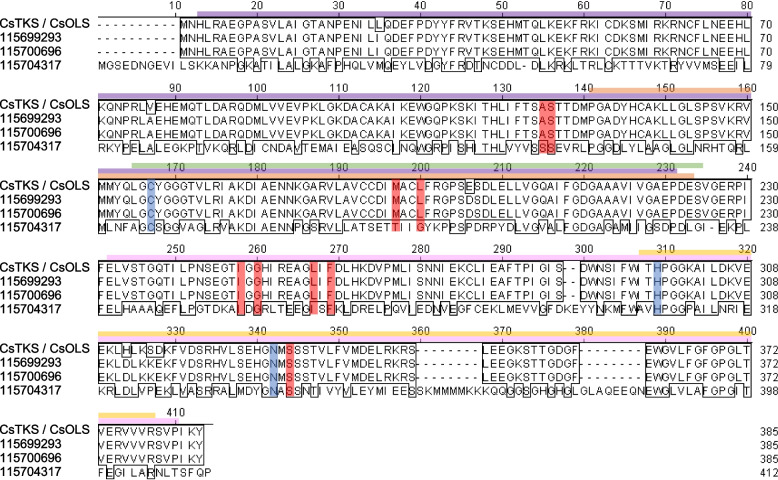
OLS/TKS orthologs of hemp Cheungsam. **A** Multiple protein sequence alignment of CsOLS/CsTKS with hemp Cheungsam orthologs. Purple bar, Chalcone and stilbene synthase N-terminal domain. Pink bar, Chalcone and stilbene synthase C-terminal domain. Green bar, 3-Oxoacyl-[acyl-carrier-protein (ACP)] synthase III domain. Yellow bar, ACP synthase C-terminal domain. Orange bar, FAE1/Type III polyketide synthase-like domain. Based on Taura et al. (2009), CHS catalytic triad residues are highlighted in blue, while residues that may be important for substrate specificity or polyketide length are highlighted in red

From the RNA-seq data set, we identified three putative *OLS/TKS* genes (unigenes *115699293*, *115700696*, and *115704317*). Among them, *115699293* and *115700696* matched 100% to each other at the amino acid sequence level (Fig. 7), but not at nucleotide sequence level (Fig. S6), indicating two copies of this gene at different loci in hemp Cheungsam. These genes also showed 98.4% amino acid sequence similarity to the database OLS/TKS (CsTKS/CsOLS; Fig. 7). On the other hand, *115704317* showed low protein homology with these sequences, with 36.6% similarity to *CsOLS* and 37.3% similarity to *115699293* and *115700696*.

Conserved domain search associated these OLS/TKS homologs with the chalcone synthase (CHS) superfamily, as N-terminal and C-terminal domains of chalcone and stilbene synthase were identified (Fig. 7). Moreover, other domains related to 3-oxoacyl-[acyl-carrier-protein (ACP)] synthase III and FAE1/Type III polyketide synthase-like protein were found in OLS/TKS homologs (Fig. 7). The identification of CHS-related domains found in OLS/TKS homologs can be explained by their high sequence similarity, as seen in the comparison between *C. sativa* OLS/TKS and *Medicago sativa* CHS (Taura et al. 2009). The comparison between CHS and OLS identified three conserved catalytic residues (Cys157, His297, Asn330; positions in CsOLS) for chain elongation and nine active site residues (Ala125, Ser126, Met187, Leu190, Ile248, Gly250, Leu257, Phe259, Ser332; positions in CsOLS) for substrate specificity (Fig. 7; Taura et al. 2009). All these residues were conserved in CsOLS as well as in unigenes *115699293* and *115700696* (Fig. 7), suggesting that *115699293* and *115700696* are *OLS/TKS* homologs in hemp Cheungsam. In contrast, the catalytic residues were conserved in *115704317* but 4 out of 9 substrate-specificity residues differ from CsOLS (Fig. 7). This suggests that while *115704317* may be catalytically similar to OLS, it likely functions as a polyketide synthase (PKS) that targets other substrates besides hexanoyl-CoA and produces polyketides of different length (Jez et al. 2000).

#### Olivetolic acid cyclase (OAC)

The linear tetraketide intermediate is further cyclized by OAC to produce OLA (Kearsey et al. 2020). Here, we identified two *OAC* orthologs (unigenes *115723437*, *115723438*) that matched 100% to the GenBank database CsOAC protein sequence (Fig. S7A). Interestingly, the nucleotide sequences of both hemp *OAC* homologs showed slight differences from the GenBank database nucleotide sequence (Fig. S7B). This suggests that hemp Cheungsam may have a single *OAC* gene with multiple transcript variants or two highly conserved genes.

CsOAC, *115723437*, and *115723438* contain the stress-responsive dimeric α + β barrel (DABB) domain (Fig. S7), which makes them structurally similar to other polyketide cyclases (Gagne et al. 2012). Enzymatic assay of DABB domain-containing OAC from *C. sativa* trichomes showed the conversion of hexanoyl-CoA to OLA, in the presence of OLS/TKS (Gagne et al. 2012). This indicates that the conserved DABB domain plays a significant role for OLA production.

#### Aromatic prenyltransferase (PT)

OLA reacts with GPP to undergo prenylation by PT, resulting in the biosynthesis of CBGA (Blatt-Janmaat and Qu 2021). The RNA-seq elucidated 7 putative PT in hemp Cheungsam. In *C. sativa*, CsPT1 and CsPT4 were previously identified to be key players in the biosynthesis of CBGA from OLA and GPP (Lim et al. 2021). In contrast, CsPT2 was categorised as a clade II PT, which was shown to be involved in tocopherol biosynthesis (Collakova and DellaPenna, 2001, Rea et al. 2019). Moreover, while CsPT3 belonged to the same phylogenetic clade as CsPT1 and CsPT4, it was demonstrated to function in Cannflavin A and B biosynthesis in *C. sativa* (Rea et al. 2019).

**Fig. 8:**
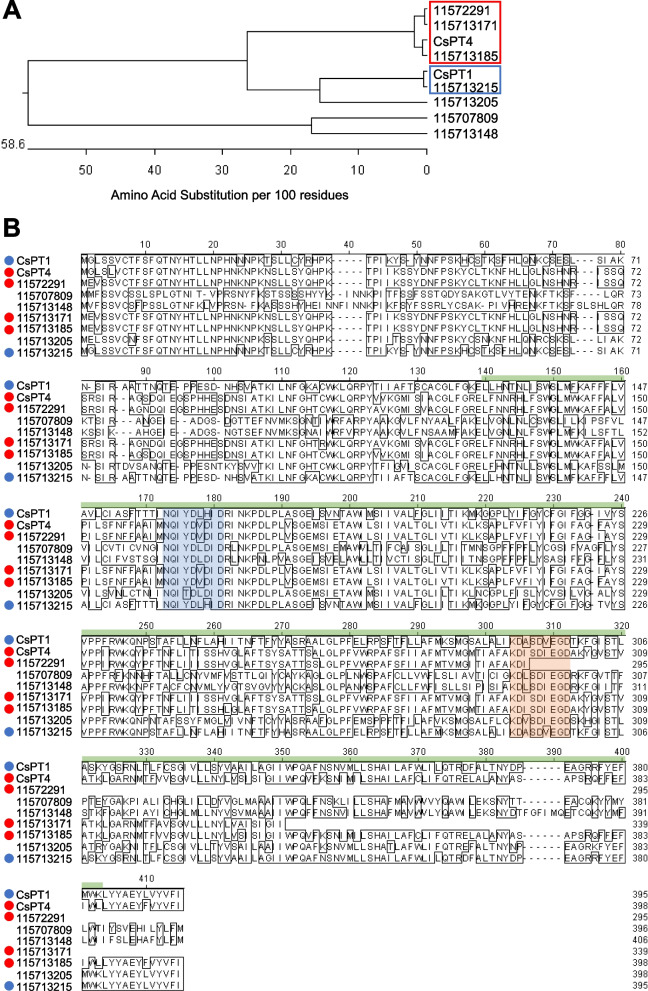
PT orthologs in hemp Cheungsam. **A** Phylogenetic tree of protein sequences of CsPT1, CsPT4, and hemp Cheungsam PT orthologs. Red and blue boxes indicate close orthologs of CsPT4 and CsPT1, respectively. **B** Multiple sequence alignment of all sequences in (A). Highlighted sequences are conserved PT motifs: NQxxDxxxD (blue), KDxxDxxGD (orange) (de Bruijn et al. 2020). Green bar, UbiA domain. CsPT1 ortholog, blue circle. CsPT4 ortholog, red circle

Phylogenetic analysis of hemp Cheungsam PT orthologs against GenBank database CsPT1 and CsPT4 revealed high protein similarity between CsPT1 with unigene *115713215* (blue box, Fig. 8A). In addition, CsPT4 formed a distinct clade with unigenes *115722991*, *115713171*, and *115713185* (red box, Fig. 8A).

Further sequence alignment was carried out for CsPT1, CsPT4, and all PT orthologs, which indicated a generally conserved UbiA prenyltransferase domain in CsPT1, CsPT4, and all high similarity orthologs (red and blue dots, Fig. 8B). The UbiA superfamily proteins are characterized as intramembrane PT that function in various biological functions such as chlorophyll biosynthesis, tocopherol biosynthesis, and secondary metabolism to produce phytoalexins and alkaloids for plant defense (Li 2016). CsPTs, which are homogentisate (HG) PTs, typically contain the conserved aspartate-rich motifs, NQxxDxxxD and KDxxDxxGD (de Bruijn et al. 2020). Interestingly, all putative PT genes in hemp Cheungsam contain the NQxxDxxxD motif (blue region, Fig. 8), but the KDxxDxxGD motif was missing from *115722991* (orange region, Fig. 8). These motifs function in regulating Mg^2+^ ions that stabilize the pyrophosphate component of prenyl donors for further reaction (de Bruijn et al. 2020). Also, PTs from the HG family have been shown to localize in the plastids (Sukumaran et al. 2018; Yang et al. 2018). As unigenes *115713171*, *115713185*, and *115713215* (Fig. 8) have high homology with CsPT1/4 and contain the conserved aspartate-rich motifs, it is possible that they are functional aromatic PTs that catalyze the conversion of OLA to CBGA in hemp Cheungsam.

### Cannabinoid oxidocyclase (CBCAS, CBDAS, THCAS)

**Fig. 9:**
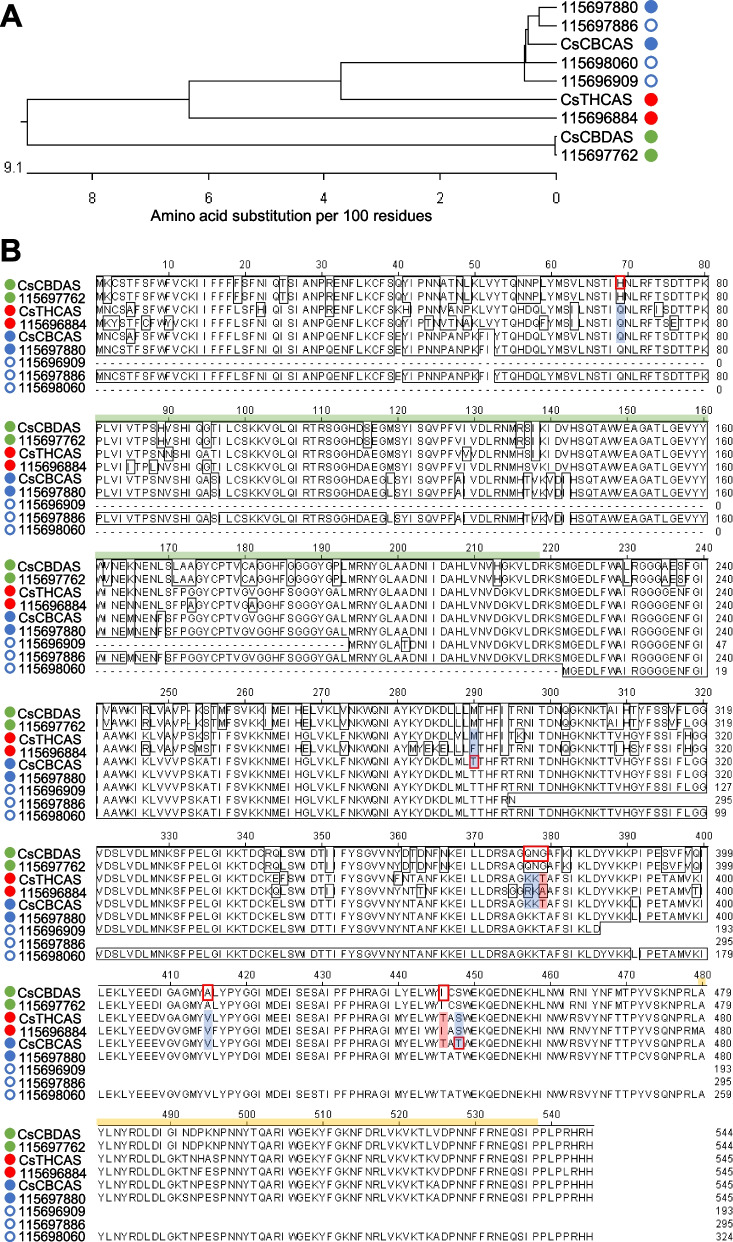
Cannabinoid oxidocyclase orthologs of hemp Cheungsam. **A** Phylogenetic tree of protein sequences of CsCBCAS, CsCBDAS, and CsTHCAS with orthologs. **B** Multiple sequence alignment of all sequences in (**A**). Green bar, FAD-binding domain. Yellow bar, BBE-like domain. Blue circle, CBCAS homolog. Green circle, CBDAS homolog. Red circle, THCAS homolog. Closed circles represent full-length sequences with stop codons. Open circles represent partial sequences

CBCAS, CBDAS, and THCAS are cannabinoid oxidocyclases that use CBGA as a substrate for the conversion to CBCA, CBDA and THCA (Jalali et al. 2019; Melzer et al. 2022). The RNA-seq data set has elucidated the following putative genes: four CBCAS, one THCAS, and one CBDAS (Fig. 9A). Multiple sequence alignment of the protein sequences showed that GenBank CsCBCAS shared high protein homology with unigenes *115696909*, *115697880*, *115697886*, and *115698060* (Fig. 9B). Moreover, CsCBDAS matched 100% to unigene *115697762* (Fig. 9A, B). As unigene *115696884* showed high similarity to both CsCBCAS (87.9%) and CsTHCAS (89.0%) (Fig. 9A), further sequence analysis was needed to elucidate its function and identity.

CBDAS, THCAS, and CBCAS are known to belong to the Berberine Bridge Enzyme (BBE)-like gene family (Sirikantaramas et al. 2004). CBDAS, THCAS, and CBCAS also feature conserved domains of the BBE-like family, including the Flavin Adenin Dinucleotide (FAD) binding domain and a C-terminal BBE-like domain (Fig. 9B; van Velzen and Schranz, 2021). These two main domains (FAD-binding domain and BBE-like domain) were found in most of cannabinoid oxidocyclase orthologs of hemp Cheungsam (Fig. 9B). However, N-terminal truncation resulted in unigenes *115696909* and *115698060* lacking the FAD-binding domain, while C-terminal truncation resulted in unigenes *115696909* and *115697886* lacking the BBE-like domain (Fig. 9B), suggesting that these unigenes are partial sequences and do not correspond to functional cannabinoid oxidocyclases. All other hemp Cheungsam cannabinoid oxidocyclase sequences were highly conserved with CBDAS, THCAS, or CBDAS, suggesting that they are full-length oxidocyclases (Fig. 9B). However, it is important to note that expression of the full-length oxidocyclase homologs were different, as unigene *115697762* (CBDAS homolog) showed more than seven-fold higher expression than unigenes *115696884* (THCAS homolog) and *115697880* (CBCAS homolog) (Fig. 5). This was consistent with the high CBDA and low THCA content of hemp Cheungsam (Moon et al. 2002).

To elucidate the identity of *115696884*, we compared specific amino acid residues at the shared active site between CsCBDAS, CsTHCAS, and CsCBCAS as identified by Lim et al. (2021). While CsCBDAS, CsTHCAS, and CsCBCAS share a generally similar amino acid sequence, specific residues at the active site may be used to differentiate between the cannabinoid oxidocyclases (red boxes, Fig. 9B; Lim et al. 2021). The multiple sequence alignment indicated that these amino acid residues in *115696884* were mostly similar to CsTHCAS, including exact matches at Gln69, Lys378, Val415, and Ser448 (blue highlights, Fig. 9B). Other residue changes in *115696884* were of similar chemical properties as CsTHCAS: Phe290 (*115696884*) was non-polar like Met290 (CsTHCAS), while Arg377 (*115696884*) had a positively charged side chain like Lys377 (CsTHCAS) (blue highlights, Fig. 9B). Besides these, *115696884* had two other residue changes that did not match CsTHCAS: Ala379 to Thr379 and Ile446 to Thr446 (red highlights, Fig. 9B). In contrast, *115696884* had three mismatches to CsCBCAS: Phe290 to Thr290, Ala379 to Thr379, and Ile446 to Thr446 (Fig. 9B). These amino acid differences suggest that unigene *115696884* may function more closely to CsTHCAS. Furthermore, the small but detectable amount of THCA in hemp Cheungsam (0.34%, Moon et al. 2002) suggests that unigene *115696884*, despite its relatively low expression in flowers (Fig. 5), may be a functional but low-activity THCAS. However, further work is required to investigate if unigene *115696884* functions as a THCAS or other cannabinoid oxidocyclase.

## Conclusion

All in all, the transcriptome analyses have shown that hemp Cheungsam expressed most cannabinoid biosynthetic pathway genes specifically in its flowers, similar to other *Cannabis* cultivars. Further investigation of each gene’s expression level suggests preferential biosynthesis of CBDA, compared to THCA and CBCA production. Moreover, sequence analysis elucidated key orthologs for each gene of the cannabinoid biosynthetic pathway in hemp Cheungsam.

## Supplementary Information

Supplementary Material 1: Supplementary Table S1. List of DEGs identified from each pairwise comparison of plant tissues (flower, leaf, stem) of hemp Cheungsam.

Supplementary Material 2: Supplementary Table S2. Genes with high or low expression in the flower, leaf, or stem.

Supplementary Material 3: Supplementary Table S3. Average FPKM of genes identified in each gene cluster.

Supplementary Material 4: Supplementary Table S4. Putative Arabidopsis orthologs of hemp Cheungsam DEGs.

Supplementary Material 5: Supplementary Table S5. Lists of GO terms from each gene cluster.

Supplementary Material 6: Supplementary Table S6. Lists of KEGG pathways from each gene cluster.

Supplementary Material 7: Supplementary Table S7. Average FPKM of cannabinoid biosynthesis pathway genes in various plant tissues (flower, leaf, stem).

Supplementary Material 8: Supplementary Figure S1. Heatmap of square of Pearson correlation coefficient. Supplementary Figure S2. KEGG pathway analysis. KEGG pathway terms were selected to have Benjamini–Hochberg adjusted *P*-value (Benjamini) < 0.05. Top 10 KEGG pathway terms are shown. Gene clusters correspond to Fig.  3B. Supplementary Figure S3. Multiple protein sequence alignment of CsAAE4, CsAAE13, and CsAAE14 with hemp Cheungsam orthologs. Green bar, AMP-binding domain. Yellow bar, AMP-binding C-terminal domain. Blue P indicates predicted peroxisomal AAE. Supplementary Figure S4. Multiple protein sequence alignment of CsAAE5 with hemp Cheungsam orthologs. Green bar, AMP-binding domain. Yellow bar, AMP-binding C-terminal domain. Blue P indicates predicted peroxisomal AAE. Supplementary Figure S5. Multiple protein sequence alignment of CsAAE7 and CsAAE9 with hemp Cheungsam orthologs. Green bar, AMP-binding domain. Yellow bar, AMP-binding C-terminal domain. Blue P indicates predicted peroxisomal AAE. Supplementary Figure S6. Multiple nucleotide sequence alignment of CsTKS/CsOLS with hemp Cheungsam ortholog genes. Supplementary Figure S7. Multiple sequence alignment of CsOAC with hemp Cheungsam orthologs. (A) Alignment of protein sequences of CsOAC with orthologs 115723437 and 115723438 . Green bar, stress-responsive dimeric α + β barrel (DABB) domain. (B) Alignment of nucleotide sequences.

## Data Availability

The data analyzed in this study are available from the corresponding authors with a reasonable request.

## Abbreviations

AAE: Acyl-activating enzyme
BLAST: Basic Local Alignment Search Tool
BP: Biological process
CBC: Cannabichromene
CBCA: Cannabichromenic acid
CBCAS: CBCA synthase
CBD: Cannabidiol
CBDA: Cannabidiolic acid
CBDAS: CBDA synthase
CBGA: Cannabigerolic acid
DAVID analysis: Database for Annotation, Visualization, and Integrated Discovery analysis
DEG: Differentially expressed genes
FPKM: Fragments per kilobase of transcript per million mapped reads
GO: Gene ontology
GPP: Geranyl pyrophosphate
GPS: GPP synthase
KEGG: Kyoto Encyclopedia of Genes and Genomes
MEP: Methylerythritol 4-phosphate
OAC: Olivetolic acid cyclase
OLA: Olivetolic acid
OLS: Olivetol synthase
PT: Aromatic prenyltransferases
RNA-seq: RNA-sequencing
THC: Δ^9^‐Tetrahydrocannabinol
THCA: Δ^9^-Tetrahydrocannabinolic acid
THCAS: THCA synthase
TKS: Tetraketide synthase
