# Supplementary material for: Genome-wide identification of cannabinoid biosynthesis genes in non-drug type Cannabis (Cannabis sativa L.) cultivar
Source: J Cannabis Res. 2024 Sep 7;6:35. doi: 10.1186/s42238-024-00246-8 (PMC11380790; doi:10.1186/s42238-024-00246-8)
Supplement: Supplementary file 8 — Supplementary Material 8: Supplementary Figure S1. Heatmap of square of Pearson correlation coefficient. Supplementary Figure S2. KEGG pathway analysis. KEGG pathway terms were selected to have Benjamini–Hochberg adjusted P-value (Benjamini) < 0.05. Top 10 KEGG pathway terms are shown. Gene clusters correspond to Fig. 3B. Supplementary Figure S3. Multiple protein sequence alignment of CsAAE4, CsAAE13, and CsAAE14 with hemp Cheungsam orthologs. Green bar, AMP-binding domain. Yellow bar, AMP-binding C-terminal domain. Blue P indicates predicted peroxisomal AAE. Supplementary Figure S4. Multiple protein sequence alignment of CsAAE5 with hemp Cheungsam orthologs. Green bar, AMP-binding domain. Yellow bar, AMP-binding C-terminal domain. Blue P indicates predicted peroxisomal AAE. Supplementary Figure S5. Multiple protein sequence alignment of CsAAE7 and CsAAE9 with hemp Cheungsam orthologs. Green bar, AMP-binding domain. Yellow bar, AMP-binding C-terminal domain. Blue P indicates predicted peroxisomal AAE. Supplementary Figure S6. Multiple nucleotide sequence alignment of CsTKS/CsOLS with hemp Cheungsam ortholog genes. Supplementary Figure S7. Multiple sequence alignment of CsOAC with hemp Cheungsam orthologs. (A) Alignment of protein sequences of CsOAC with orthologs 115723437 and 115723438 . Green bar, stress-responsive dimeric α + β barrel (DABB) domain. (B) Alignment of nucleotide sequences. [file 42238_2024_246_MOESM8_ESM.pptx]

## Slide 1
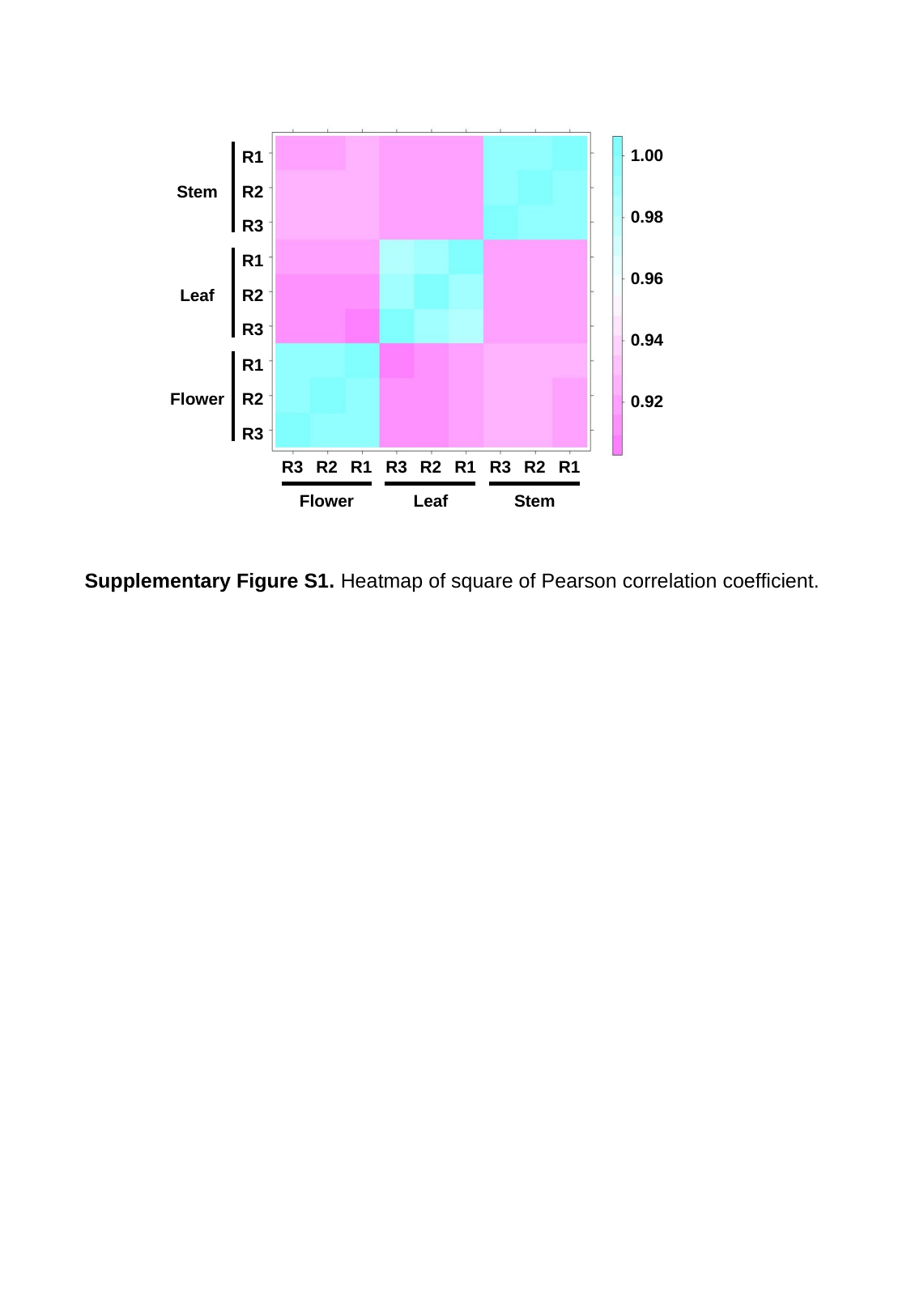

1.00
R1
Stem
R2
0.98
R3
R1
0.96
Leaf
R2
R3
0.94
R1
Flower
R2
0.92
R3
R3
R2
R1
R3
R2
R1
R3
R2
R1
Flower
Leaf
Stem
Supplementary Figure S1. Heatmap of square of Pearson correlation coefficient.

## Slide 2
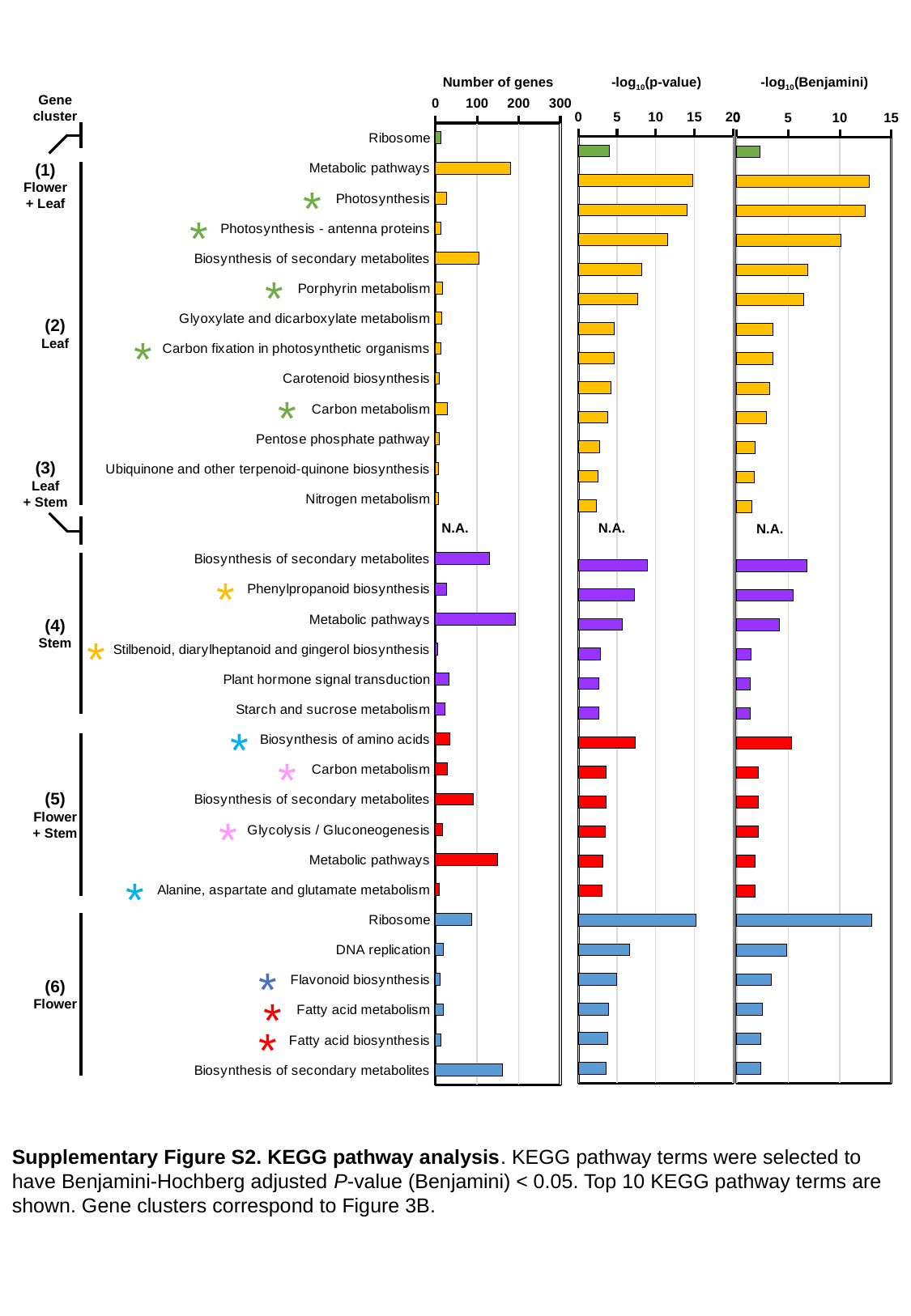

Number of genes
-log10(p-value)
-log10(Benjamini)
Gene
cluster
### Chart
| Category | |
|---|---|
| Ribosome | 4.000153138579865 |
| Metabolic pathways | 14.786112433768647 |
| Photosynthesis | 14.122336097302638 |
| Photosynthesis - antenna proteins | 11.60442428423051 |
| Biosynthesis of secondary metabolites | 8.246562873488358 |
| Porphyrin metabolism | 7.726758467018807 |
| Glyoxylate and dicarboxylate metabolism | 4.682499910404345 |
| Carbon fixation in photosynthetic organisms | 4.619810693344253 |
| Carotenoid biosynthesis | 4.268460518448365 |
| Carbon metabolism | 3.836305060853379 |
| Pentose phosphate pathway | 2.7244562944960156 |
| Ubiquinone and other terpenoid-quinone biosynthesis | 2.591217354006224 |
| Nitrogen metabolism | 2.342897591989348 |
| | None |
| Biosynthesis of secondary metabolites | 8.881933068557746 |
| Phenylpropanoid biosynthesis | 7.234322048159476 |
| Metabolic pathways | 5.73524031996656 |
| Stilbenoid, diarylheptanoid and gingerol biosynthesis | 2.8257542908468007 |
| Plant hormone signal transduction | 2.6381691619733725 |
| Starch and sucrose metabolism | 2.6320765990575405 |
| Biosynthesis of amino acids | 7.366552697263532 |
| Carbon metabolism | 3.6131316108942575 |
| Biosynthesis of secondary metabolites | 3.5901232057845327 |
| Glycolysis / Gluconeogenesis | 3.530786255126989 |
| Metabolic pathways | 3.1780543025146333 |
| Alanine, aspartate and glutamate metabolism | 3.0421916422429645 |
| Ribosome | 15.18140042238857 |
| DNA replication | 6.598019915643684 |
| Flavonoid biosynthesis | 4.987256130370659 |
| Fatty acid metabolism | 3.9479471782031577 |
| Fatty acid biosynthesis | 3.7531385765820993 |
| Biosynthesis of secondary metabolites | 3.6017877287619253 |
### Chart
| Category | |
|---|---|
| Ribosome | 2.222001888196221 |
| Metabolic pathways | 12.831869924329322 |
| Photosynthesis | 12.469123583527294 |
| Photosynthesis - antenna proteins | 10.127303029510848 |
| Biosynthesis of secondary metabolites | 6.894380355376995 |
| Porphyrin metabolism | 6.4714859619155005 |
| Glyoxylate and dicarboxylate metabolism | 3.5106662239191864 |
| Carbon fixation in photosynthetic organisms | 3.5106662239191864 |
| Carotenoid biosynthesis | 3.2173079960009847 |
| Carbon metabolism | 2.836305060853379 |
| Pentose phosphate pathway | 1.7702137850566912 |
| Ubiquinone and other terpenoid-quinone biosynthesis | 1.6783675297251253 |
| Nitrogen metabolism | 1.4678363285976477 |
| | None |
| Biosynthesis of secondary metabolites | 6.80275182251012 |
| Phenylpropanoid biosynthesis | 5.456170797775832 |
| Metabolic pathways | 4.1331803286385975 |
| Stilbenoid, diarylheptanoid and gingerol biosynthesis | 1.3486330361271373 |
| Plant hormone signal transduction | 1.3310466033935586 |
| Starch and sucrose metabolism | 1.3310466033935586 |
| Biosynthesis of amino acids | 5.29836683551737 |
| Carbon metabolism | 2.0646603847087888 |
| Biosynthesis of secondary metabolites | 2.0646603847087888 |
| Glycolysis / Gluconeogenesis | 2.0646603847087888 |
| Metabolic pathways | 1.8088384451044914 |
| Alanine, aspartate and glutamate metabolism | 1.7521570308804482 |
| Ribosome | 13.091495310949172 |
| DNA replication | 4.809144799868268 |
| Flavonoid biosynthesis | 3.374472273650921 |
| Fatty acid metabolism | 2.460102058091721 |
| Fatty acid biosynthesis | 2.3622034694787195 |
| Biosynthesis of secondary metabolites | 2.2900338677061707 |
### Chart
| Category | |
|---|---|
| Ribosome | 13.0 |
| Metabolic pathways | 181.0 |
| Photosynthesis | 26.0 |
| Photosynthesis - antenna proteins | 14.0 |
| Biosynthesis of secondary metabolites | 105.0 |
| Porphyrin metabolism | 16.0 |
| Glyoxylate and dicarboxylate metabolism | 15.0 |
| Carbon fixation in photosynthetic organisms | 14.0 |
| Carotenoid biosynthesis | 9.0 |
| Carbon metabolism | 29.0 |
| Pentose phosphate pathway | 10.0 |
| Ubiquinone and other terpenoid-quinone biosynthesis | 8.0 |
| Nitrogen metabolism | 8.0 |
| | None |
| Biosynthesis of secondary metabolites | 129.0 |
| Phenylpropanoid biosynthesis | 27.0 |
| Metabolic pathways | 193.0 |
| Stilbenoid, diarylheptanoid and gingerol biosynthesis | 5.0 |
| Plant hormone signal transduction | 32.0 |
| Starch and sucrose metabolism | 22.0 |
| Biosynthesis of amino acids | 35.0 |
| Carbon metabolism | 29.0 |
| Biosynthesis of secondary metabolites | 91.0 |
| Glycolysis / Gluconeogenesis | 17.0 |
| Metabolic pathways | 149.0 |
| Alanine, aspartate and glutamate metabolism | 10.0 |
| Ribosome | 87.0 |
| DNA replication | 18.0 |
| Flavonoid biosynthesis | 12.0 |
| Fatty acid metabolism | 19.0 |
| Fatty acid biosynthesis | 14.0 |
| Biosynthesis of secondary metabolites | 162.0 |(1)
Flower
+ Leaf
*
*
*
(2)
Leaf
*
*
(3)
Leaf
+ Stem
N.A.
N.A.
N.A.
*
(4)
Stem
*
*
*
(5)
Flower
+ Stem
*
*
*
(6)
Flower
*
*
Supplementary Figure S2. KEGG pathway analysis. KEGG pathway terms were selected to have Benjamini-Hochberg adjusted P-value (Benjamini) < 0.05. Top 10 KEGG pathway terms are shown. Gene clusters correspond to Figure 3B.

## Slide 3
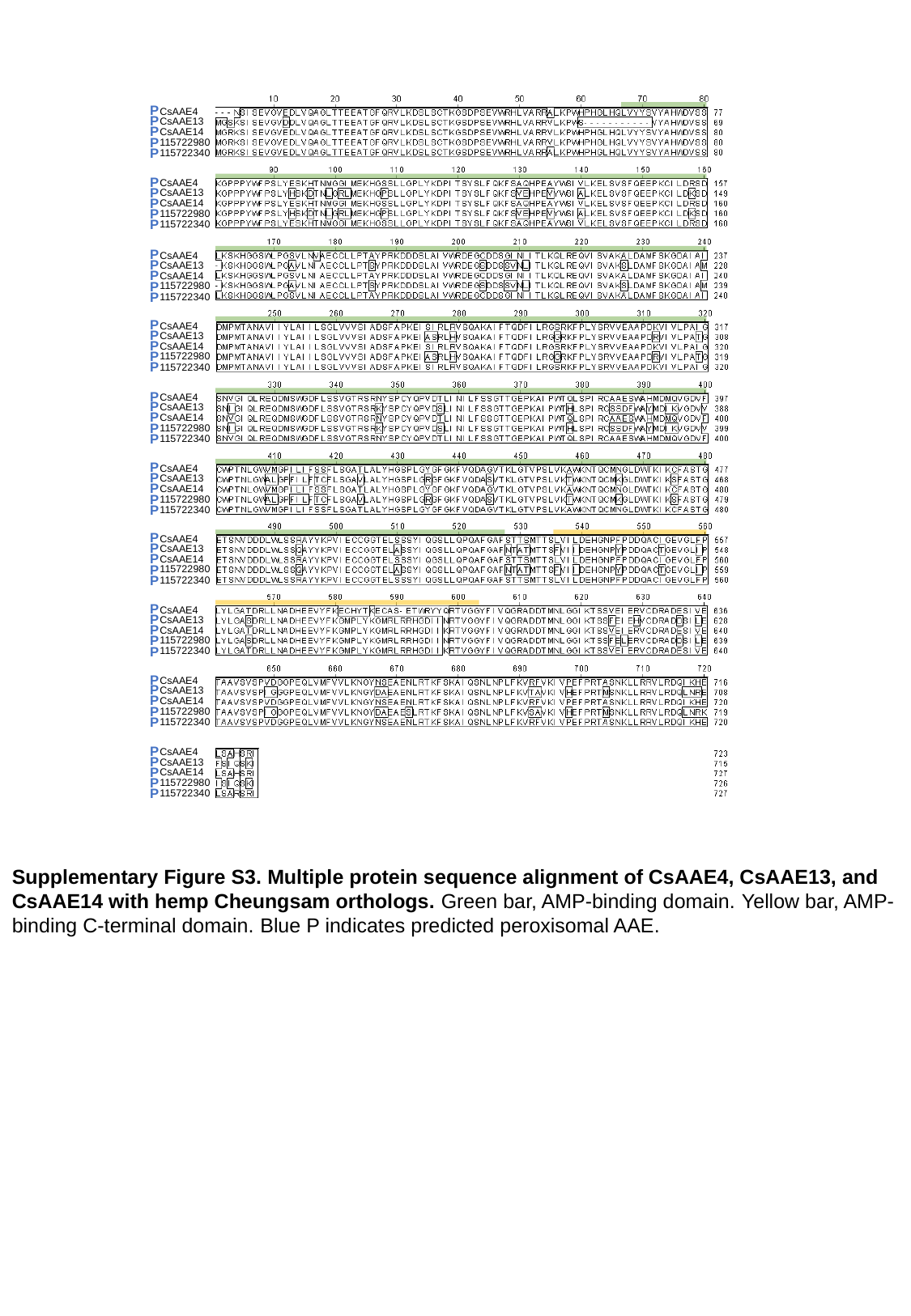

P
CsAAE4
CsAAE13
CsAAE14
115722980
115722340
P
P
P
P
P
CsAAE4
CsAAE13
CsAAE14
115722980
115722340
P
P
P
P
P
CsAAE4
CsAAE13
CsAAE14
115722980
115722340
P
P
P
P
P
CsAAE4
CsAAE13
CsAAE14
115722980
115722340
P
P
P
P
P
CsAAE4
CsAAE13
CsAAE14
115722980
115722340
P
P
P
P
P
CsAAE4
CsAAE13
CsAAE14
115722980
115722340
P
P
P
P
P
CsAAE4
CsAAE13
CsAAE14
115722980
115722340
P
P
P
P
P
CsAAE4
CsAAE13
CsAAE14
115722980
115722340
P
P
P
P
P
CsAAE4
CsAAE13
CsAAE14
115722980
115722340
P
P
P
P
P
CsAAE4
CsAAE13
CsAAE14
115722980
115722340
P
P
P
P
Supplementary Figure S3. Multiple protein sequence alignment of CsAAE4, CsAAE13, and CsAAE14 with hemp Cheungsam orthologs. Green bar, AMP-binding domain. Yellow bar, AMP-binding C-terminal domain. Blue P indicates predicted peroxisomal AAE.

## Slide 4
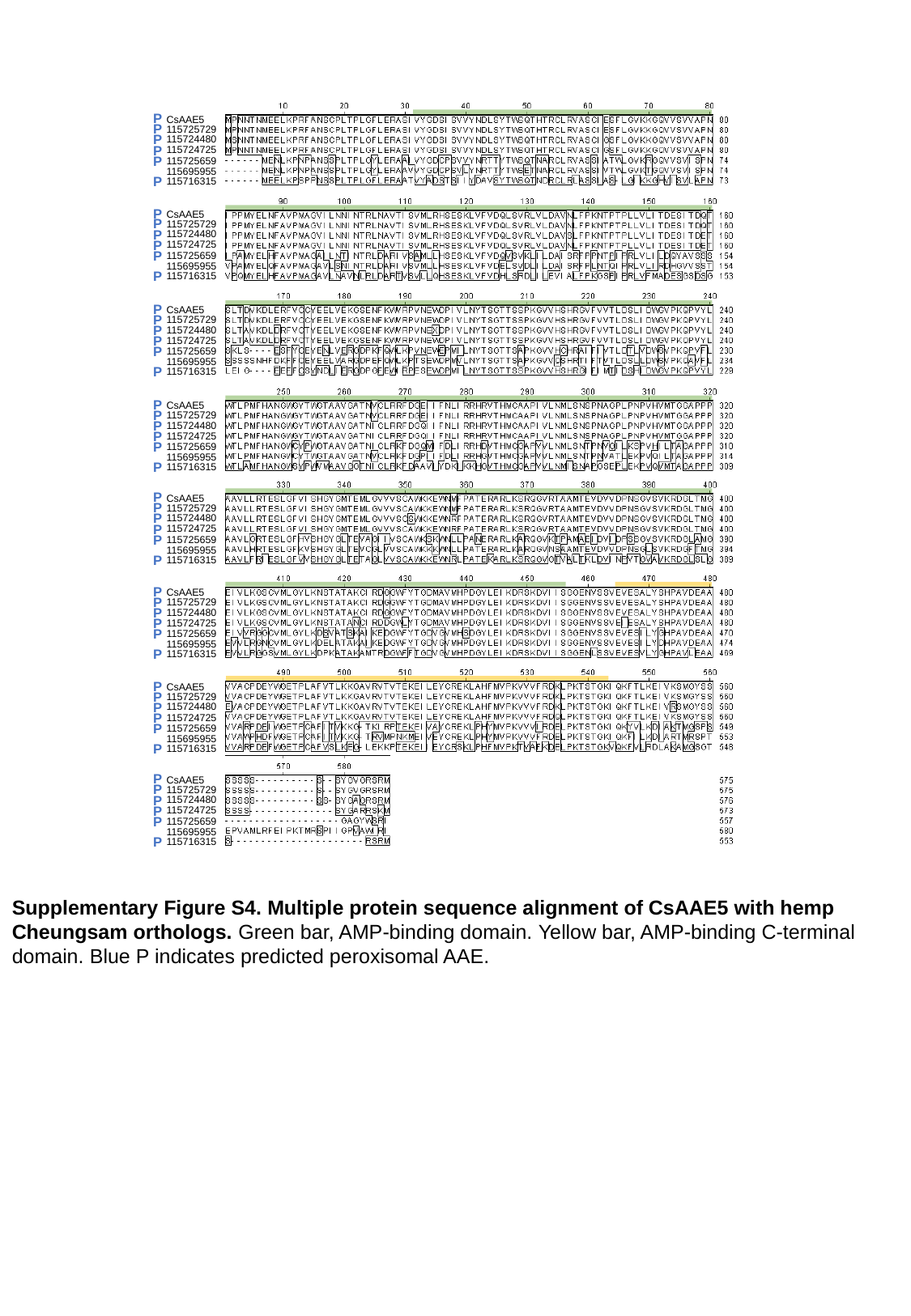

P
CsAAE5
P
115725729
P
115724480
P
115724725
P
115725659
115695955
P
115716315
P
CsAAE5
P
115725729
P
115724480
P
115724725
P
115725659
115695955
P
115716315
P
CsAAE5
P
115725729
P
115724480
P
115724725
P
115725659
115695955
P
115716315
P
CsAAE5
P
115725729
P
115724480
P
115724725
P
115725659
115695955
P
115716315
P
CsAAE5
P
115725729
P
115724480
P
115724725
P
115725659
115695955
P
115716315
P
CsAAE5
P
115725729
P
115724480
P
115724725
P
115725659
115695955
P
115716315
P
CsAAE5
P
115725729
P
115724480
P
115724725
P
115725659
115695955
P
115716315
P
CsAAE5
P
115725729
P
115724480
P
115724725
P
115725659
115695955
P
115716315
Supplementary Figure S4. Multiple protein sequence alignment of CsAAE5 with hemp Cheungsam orthologs. Green bar, AMP-binding domain. Yellow bar, AMP-binding C-terminal domain. Blue P indicates predicted peroxisomal AAE.

## Slide 5
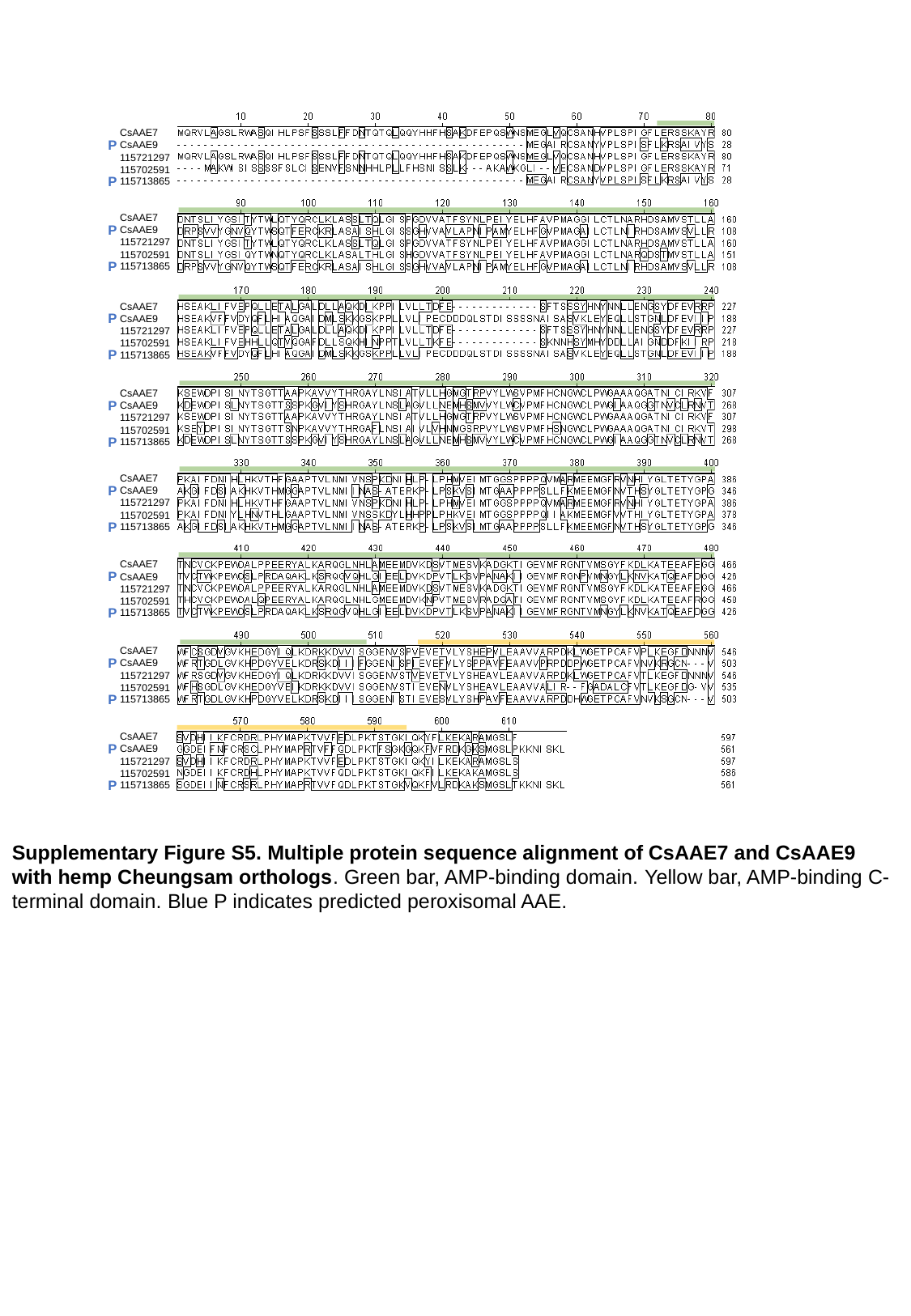

CsAAE7
CsAAE9
115721297
115702591
115713865
P
P
CsAAE7
CsAAE9
115721297
115702591
115713865
P
P
CsAAE7
CsAAE9
115721297
115702591
115713865
P
P
CsAAE7
CsAAE9
115721297
115702591
115713865
P
P
CsAAE7
CsAAE9
115721297
115702591
115713865
P
P
CsAAE7
CsAAE9
115721297
115702591
115713865
P
P
CsAAE7
CsAAE9
115721297
115702591
115713865
P
P
CsAAE7
CsAAE9
115721297
115702591
115713865
P
P
Supplementary Figure S5. Multiple protein sequence alignment of CsAAE7 and CsAAE9 with hemp Cheungsam orthologs. Green bar, AMP-binding domain. Yellow bar, AMP-binding C-terminal domain. Blue P indicates predicted peroxisomal AAE.

## Slide 6
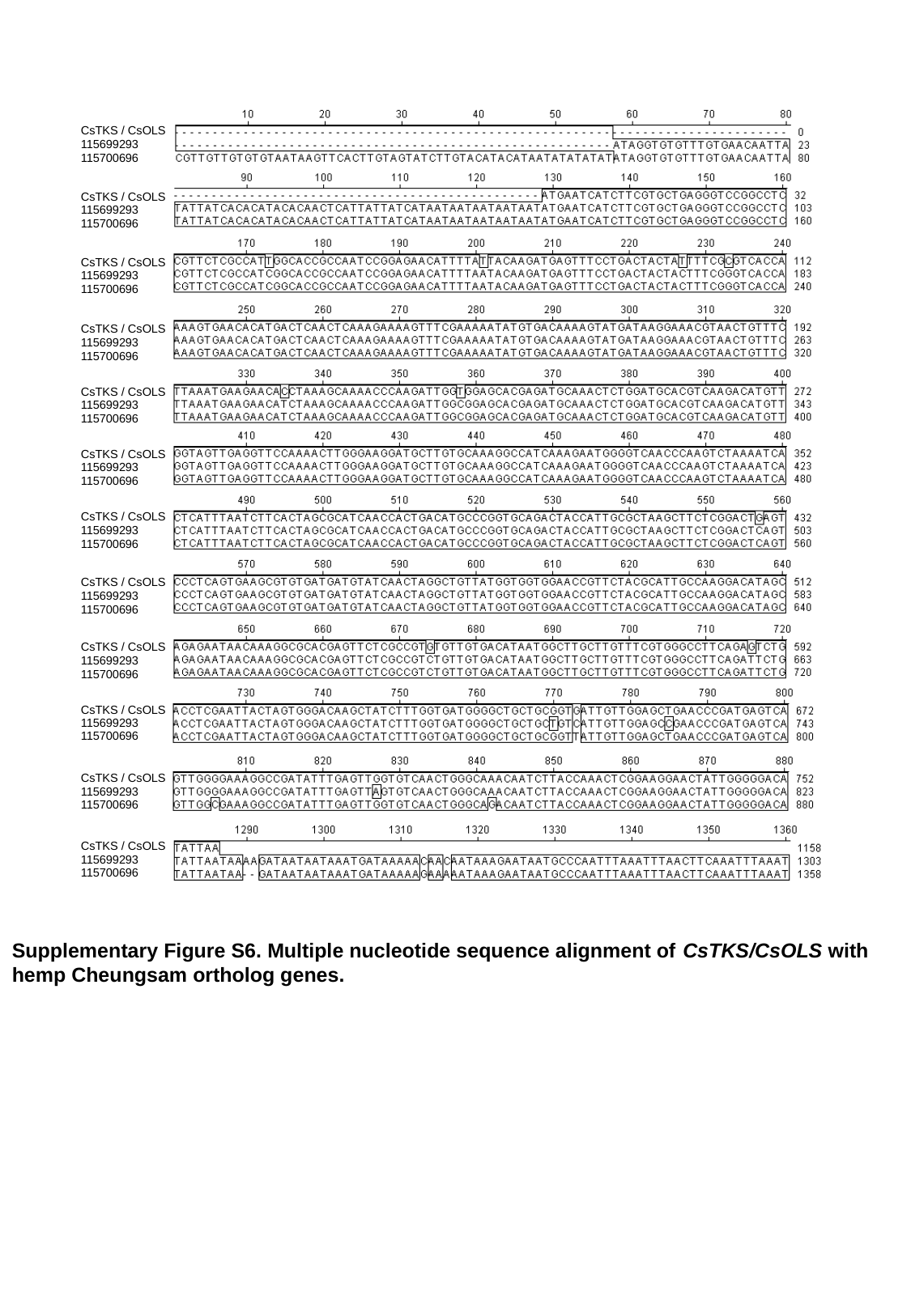

CsTKS / CsOLS
115699293
115700696
CsTKS / CsOLS
115699293
115700696
CsTKS / CsOLS
115699293
115700696
CsTKS / CsOLS
115699293
115700696
CsTKS / CsOLS
115699293
115700696
CsTKS / CsOLS
115699293
115700696
CsTKS / CsOLS
115699293
115700696
CsTKS / CsOLS
115699293
115700696
CsTKS / CsOLS
115699293
115700696
CsTKS / CsOLS
115699293
115700696
CsTKS / CsOLS
115699293
115700696
CsTKS / CsOLS
115699293
115700696
Supplementary Figure S6. Multiple nucleotide sequence alignment of CsTKS/CsOLS with hemp Cheungsam ortholog genes.

## Slide 7
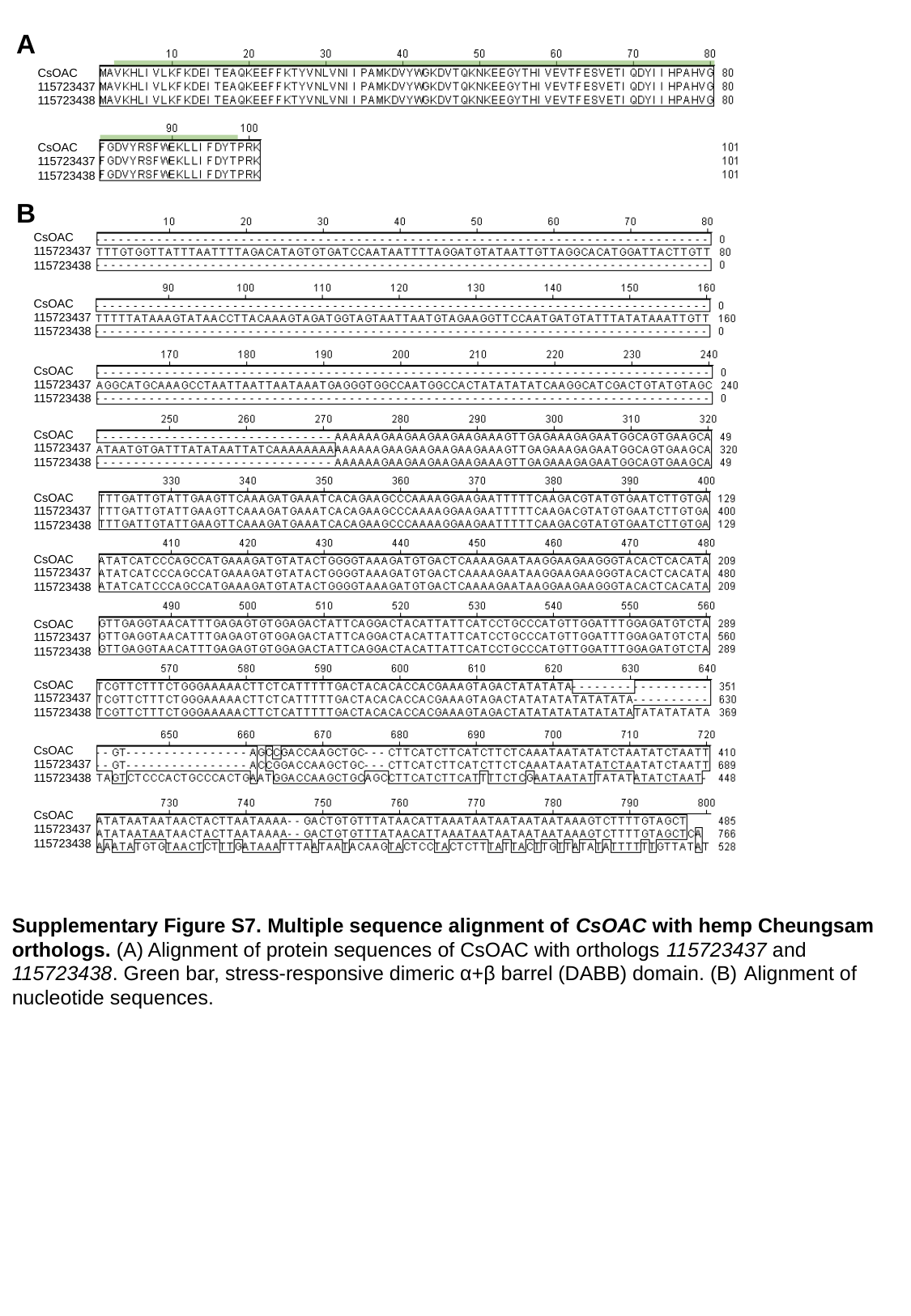

A
CsOAC
115723437
115723438
CsOAC
115723437
115723438
B
CsOAC
115723437
115723438
CsOAC
115723437
115723438
CsOAC
115723437
115723438
CsOAC
115723437
115723438
CsOAC
115723437
115723438
CsOAC
115723437
115723438
CsOAC
115723437
115723438
CsOAC
115723437
115723438
CsOAC
115723437
115723438
CsOAC
115723437
115723438
Supplementary Figure S7. Multiple sequence alignment of CsOAC with hemp Cheungsam orthologs. (A) Alignment of protein sequences of CsOAC with orthologs 115723437 and 115723438. Green bar, stress-responsive dimeric α+β barrel (DABB) domain. (B) Alignment of nucleotide sequences.
